# Investigation of drug release modulation from poly(2-oxazoline) micelles through ultrasound

**DOI:** 10.1038/s41598-018-28140-3

**Published:** 2018-07-02

**Authors:** Alice Rita Salgarella, Anna Zahoranová, Petra Šrámková, Monika Majerčíková, Ewa Pavlova, Robert Luxenhofer, Juraj Kronek, Igor Lacík, Leonardo Ricotti

**Affiliations:** 1 The BioRobotics Institute, Scuola Superiore Sant’Anna, Viale R. Piaggio 34, 56025 Pontedera (Pisa), Italy; 2 Department for Biomaterials Research, Polymer Institute of the Slovak Academy of Sciences, Dúbravská cesta 9, 845 41 Bratislava, Slovakia; 3 Institute of Natural and Synthetic Polymers, Faculty of Chemical and Food Technology, Slovak University of Technology, Radlinského 9, 812 37 Bratislava, Slovakia; 4 Institute of Macromolecular Chemistry, Academy of Sciences of the Czech Republic, Heyrovského nám. 2, 162 06 Prague 6, Czech Republic; 5 Functional Polymer Materials, Chair for Chemical Technology of Materials Synthesis, University of Würzburg, Röntgenring 11, 97070 Würzburg, Germany

## Abstract

Among external stimuli used to trigger release of a drug from a polymeric carrier, ultrasound has gained increasing attention due to its non-invasive nature, safety and low cost. Despite this attention, there is only limited knowledge about how materials available for the preparation of drug carriers respond to ultrasound. This study investigates the effect of ultrasound on the release of a hydrophobic drug, dexamethasone, from poly(2-oxazoline)-based micelles. Spontaneous and ultrasound-mediated release of dexamethasone from five types of micelles made of poly(2-oxazoline) block copolymers, composed of hydrophilic poly(2-methyl-2-oxazoline) and hydrophobic poly(2-*n*-propyl-2-oxazoline) or poly(2-butyl-2-oxazoline-co-2-(3-butenyl)-2-oxazoline), was studied. The release profiles were fitted by zero-order and Ritger-Peppas models. The ultrasound increased the amount of released dexamethasone by 6% to 105% depending on the type of copolymer, the amount of loaded dexamethasone, and the stimulation time point. This study investigates for the first time the interaction between different poly(2-oxazoline)-based micelle formulations and ultrasound waves, quantifying the efficacy of such stimulation in modulating dexamethasone release from these nanocarriers.

## Introduction

Triggerable drug release systems based on stimuli-responsive polymers are emerging as platforms aiming at increasing the effectiveness and reducing the side effects of traditional therapies^1^. Such on-demand targeted therapy may be based on nanocarriers^2^, thin films and hydrogels^3^, nanogels^4^, and other miniaturized systems applied for several pathological conditions. Such pathologies include nervous system disorders, bone inflammation and infections, chronic pain, oncological diseases, and diabetes^5,6^.

Polymeric carriers can be designed in order to respond to different stimuli (chemical and physical ones), so to somewhat preferentially deliver the desired drug at the site of interest. Such stimuli can be internal to the body, such as pH changes, redox gradients, enzymes action, temperature changes^7^. In this case, the triggering action is determined by local alterations of the diseased tissues with respect to the healthy ones. Stimuli can be also external to the body: this makes a remote triggering possible, thus adding a degree of controllability to the therapy. The remote drug release triggering can be based on different driving physical inputs, including electric and magnetic fields, light and ultrasound^8^.

Among these external stimuli, ultrasound (US) possesses several advantages^9,10^: (1) US penetrates through a number of different tissues, especially soft ones, in a safe and reliable way without a dramatic energy dissipation, (2) US is a versatile tool able to trigger both mechanical and thermal phenomena, and (3) US has promising theranostic abilities, being an effective tool for enhancing drug release and visualizing the target during the therapeutic action. Most importantly, it is cheap and readily available world-wide. Consequently, US stimulation raises interest in the biomedical community, but the mechanism of US waves interaction with nanocarriers for controlled delivery of therapeutic agents remain largely to be elucidated^11^.

Nanocapsules, microbubbles, liposomes, and micelles are typical carriers developed for this purpose^10^. In all these systems, the polymeric structure is permanently or temporarily perturbed by mechanical and/or thermal action of US, resulting in a modulated drug delivery^12^. Arguably, polymer micelles are particularly interesting since they can be easily prepared by self-assembling of block copolymers^13^. Their small size range (diameter typically between 10 and 100 nm) is ideal to enhance penetration into target tissue and decrease renal excretion, allowing some sort of passive targeting^12^. Some examples of US-triggered micelles used for drug release have been recently reviewed^10,14,15^. The US-mediated release of a drug from a micellar core proceeds either (1) irreversibly, e.g. via ester hydrolysis induced typically by high intensity focused ultrasound (HIFU, 1.1 MHz)^16,17^, or (2) reversibly via transient cavitation effect of low-frequency ultrasound (20–90 kHz)^18–20^. In view of recent achievements, it appears beneficial to investigate the ability for US-mediated drug release for already well-established micellar systems based on more versatile materials with variable hydrophobicity, higher drug loading capacity and stealth properties. Poly(2-oxazoline)s (POx) are a family of polymers featuring these attributes.

POx are synthetic polymers, prepared by living cationic ring-opening polymerization of 2-oxazolines. The living nature of the polymerization process enables to control molar mass, dispersity, and overall architecture of resulting polymers^21^. Moreover, it allows to prepare well-defined block copolymers by a simple subsequent addition of properly selected monomers^22^. A large library of 2-oxazoline monomers, including hydrophilic, thermoresponsive and hydrophobic ones, allows for forming versatile POx structures that exhibit versatile chemical and physical characteristics. Hydrophilic and amphiphilic POx exhibit stealth behavior^23^ and have been found to be non-toxic both *in vitro*^24,25^ and *in vivo*^26^. For these reasons, they are attracting an increasing attention in different biomedical fields, including the preparation of drug^27^ and protein^28,29^ conjugates, anti-fouling^30^ and thermoresponsive^31^ membranes, hydrogels^32,33^, and vectors for gene^34,35^ and radionuclide^36^ delivery.

It has been reported that amphiphilic block and gradient copolymers of (2-oxazoline)s self-assemble in aqueous solution, resulting in formation of polymeric micelles^37,38^, polymersomes^39^, and vesicles^40^. The hydrophobic core of such self-assembled aggregates can be used for a confinement of hydrophobic drugs. As recently demonstrated^41–43^, triblock copoly(2-oxazoline)s comprising poly(2-butyl-2-oxazoline) are able to solubilize large amounts, more than 40 wt%, of anticancer drugs. The performance of POx-based drug delivery systems, including micelles, is highly dependent on the chemical composition of both the copolymer and the drug^44–46^. This factor is significantly under-investigated, since most of studies in this field focus on a single condition used for micelle formulation in combination with a single drug type. Thus, a systematic investigation of different POx types, based on different comonomers and chain lengths, is crucial for specific drug delivery applications.

This paper represents a first study to investigate the effect of a low-frequency ultrasound on the drug release from POx-based polymeric micelles. To this purpose, five different amphiphilic block copoly(2-oxazoline)s composed of poly(2-methyl-2-oxazoline) (MetOx) as a hydrophilic part and poly(2-*n*-propyl-2-oxazoline) (nPropOx) or poly(2-butyl-2-oxazoline-co-2-(3-butenyl)-2-oxazoline) ((ButOx-co-EnOx)) as a hydrophobic part were prepared and characterized. These copolymers differed in the block sequence, i.e. represented diblock and triblock architectures. Their ability to encapsulate dexamethasone (Dex), an anti-inflammatory hydrophobic drug, was assessed by means of high performance liquid chromatography (HPLC). The stability of Dex-loaded micelles in PBS was determined by a dynamic light scattering (DLS). Subsequently, drug release measurements were performed with and without US stimulation. The release profiles were evaluated and fitted by different mathematical models.

## Results and Discussion

**Figure 1:**
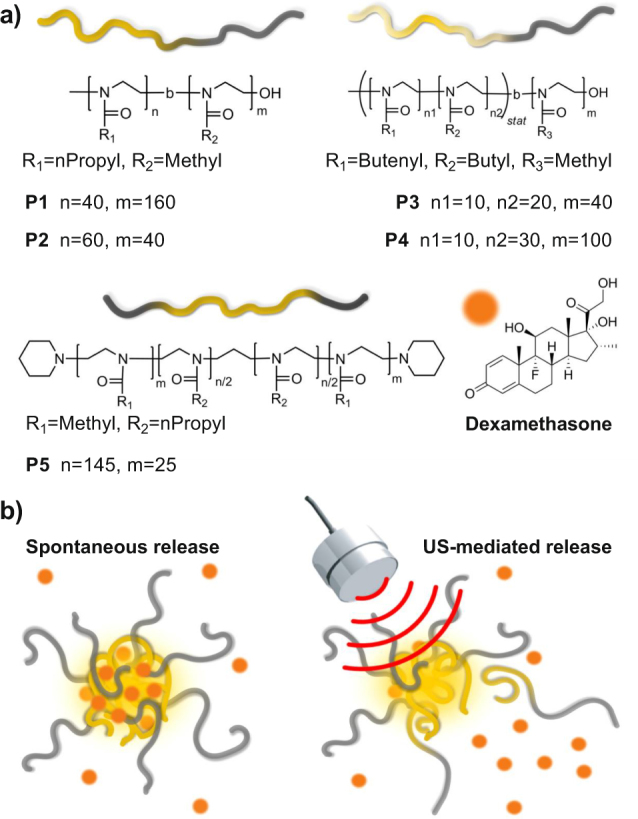
Representation of ultrasound triggered release from different POx micelle types. (**a**) Chemical structures and illustrative images of diblock and triblock copolymers P1–P5 (yellow: hydrophobic block, grey: hydrophilic block), and of dexamethasone used for drug release studies. (**b**) Depiction of spontaneous release (left) and ultrasonic-mediated release (right) from POx micelles.

For the study of spontaneous and ultrasound-mediated release of Dex hydrophobic drug, we prepared five different POx-based amphiphilic block copolymers shown in Fig. 1a. P1 (nPropOx_40_-MetOx_160_) and P2 (nPropOx_60_-MetOx_40_) are diblock copolymers consisting of MetOx and nPropOx, which differ in their chain length and blocks ratio. P3 ((EnOx_10_-co-ButOx_20_)-b-(MetOx_40_)) and P4 ((EnOx_10_-co-ButOx_30_)-b-(MetOx_100_)) are diblock terpolymers, with a hydrophobic part containing ButOx and EnOx. It is well established that EnOx allow further modifications, *e*.*g*., covalent attachment of drugs or targeting moieties^47^, and crosslinking of micellar core^48^. This feature has not been utilized in the current study but opens the possibility for further modifications in the follow-up studies. Finally, P5 (MetOx_25_-b-nPropOx_145_-b-MetOx_25_) is a triblock copolymer consisting of a central nPropOx and two flanking hydrophilic MetOx parts. This architecture was inspired by Pluronics, the well-known triblock copolymers composed of a thermoresponsive poly(propylene glycol) inner block and two outer hydrophilic poly(ethylene glycol) blocks, previously explored in the US-mediated drug delivery^14,49^.

The block copolymers were characterized by means of nuclear magnetic resonance (NMR) spectroscopy and size exclusion chromatography (SEC). The results are presented in Supplementary Table S1. SEC elugrams are shown in Supplementary Fig. S20. Except for the copolymer P1, which showed the highest hydrophilic fraction, all other copolymers exhibited an increase of the experimental hydrophilic molar fraction, *F*_exp_, in comparison to theoretical values *F*_theor_. This observation is in agreement with our recent experience^50^, in which a discrepancy between theoretical and experimental composition of triblock copolymers made of MetOx and nPropOx was found, also in favor of a hydrophilic fraction. Such discrepancy can be explained by an inefficient precipitation of copolymers in diethyl ether during a purification step, which led to the fractionation of the sample. This was proven by the presence of a more hydrophobic copolymer fraction remaining in the diethyl ether fraction. This is readily reasoned by the highly amphiphilic character of POx with short side chains such as nPropOx. This results in an increase of a hydrophilic copolymer fraction in the resulting copolymers used for micelle preparation. The Supplementary Table S1 demonstrates that a higher fraction of a hydrophobic monomer in the copolymer is consequently responsible for a higher material loss. This table also contains the information on experimental *M*_n,exp_ values determined by SEC in DMF. For most of copolymers, the *M*_n,exp_ values do not significantly differ from *M*_n,theor_. However, these data should be considered only as indicative. The reasons are (1) an incomplete precipitation of copolymers described above, and (2) the calibration relative to polystyrene standards, that is unlikely to represent an equivalent for all copolymers of very different chemical composition. However, in the absence of adequate calibration standards, the current approach appears reasonably suitable. In addition, also absolute technique such as SEC with light scattering detector, provides inadequate information on molar masses of POx possessing shorter polymer chains, as shown recently by Schubert and Nischang^51^.

**Figure 2:**
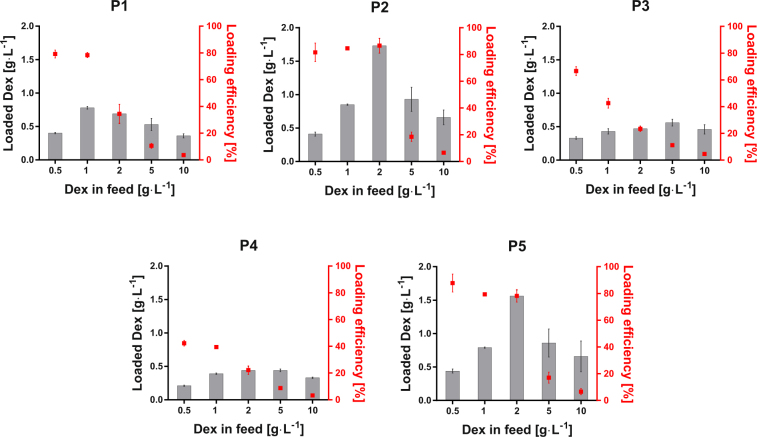
Loading of Dex in micelles expressed as loaded mass and loading efficiency. Double scaled plots representing the loaded Dex in poly(2-oxazoline)-based micelles with grey columns (means ± standard deviation (SD), n = 4), and corresponding loading efficiency with red dots (means ± SD, n = 4). P1 (nPropOx_40_-MetOx_160_); P2 (nPropOx_60_-MetOx_40_); P3 (EnOx_10_-ButOx_20_)_stat_-b-MetOx_40_); P4 (EnOx_10_-ButOx_30_)_stat_-b- MetOx_100_); P5 (MetOx_25_-nPropOx_145_-MetOx_25_). The loading was measured in water by high performance liquid chromatography, using a constant copolymer concentration of 10 g∙L^−1^.

The capability of prepared block copolymers to encapsulate hydrophobic compounds was tested using Dex as a model hydrophobic drug of a limited water-solubility (0.089 g∙L^−1^ at 25 °C^52^). The polymeric micelles with encapsulated Dex were prepared by thin film re-hydration method, which, as a simple and fast procedure, has been previously used for encapsulation of several water insoluble compounds in POx micelles^41,42,46^. Figure 2 shows the Dex loading data for the different polymeric micelles, expressed as loaded Dex concentration and loading efficiency (loaded Dex mass vs Dex in feed), obtained by HPLC. The polymer concentration was fixed in this study at 10 g∙L^−1^. This arbitrary value was inspired by previous reports on similar systems^43,46^. It should also be evidenced that our experimental protocol cannot remove free Dex contained in the solution. Therefore, we must assume that 0.089 g∙L^−1^ free Dex is always present in the solution. The micelles exhibited large variations in loading efficiency depending on their chemical structure. On the one hand, diblock copolymers composed of nPropOx exhibited loading efficiencies around 80% up to a Dex feed of 1 g∙L^−1^ (P1) and 2 g∙L^−1^ (P2). On the other hand, diblock copolymers containing EnOx-co-ButOx as hydrophobic block (P3 and P4) featured by a considerably lower loading efficiency in the whole range of Dex concentrations. This is surpising, as EnOx-co-ButOx is supposed to create a much more hydrophobic microenvironment compared to PropOx. In particular, for the lowest studied feed concentration of Dex, *i*.*e*., 0.5 g∙L^−1^, the measured loading efficiency for P3 was 67%, while for P4 it was only 42%. The triblock copolymer P5 exhibited relatively high loading efficiencies up to a Dex concentration of 2 g∙L^−1^, comparable with the results obtained for the copolymer P2. Both copolymers with the highest loading efficiencies also exhibit the lowest hydrophilic fractions *F*_exp_ (51 and 43% for P2 and P5, respectively) compared to the other copolymers. These data suggest that the loading efficiency depends on the hydrophilic fraction as well as on the chemical structure of a hydrophobic comonomer in a non-trivial manner.

The loading capacity is another characteristic of micellar systems, used as drug carriers, expressed as the ratio between the mass of loaded drug and the total mass of micelles containing loaded drug (please refer to Methods section). The maximal loading capacities of the prepared polymeric micelles P1–P5, as well as other micellar formulations reported in the literature, are shown in Supplementary Fig. S1. They ranged from 4.2% for P4 to 14.7% for P2. In comparison to our systems, other polymeric micelles recently used for Dex encapsulation exhibited lower loading capacities. A loading capacity in the range 0.5–3% was observed for Dex-loaded micelles composed of poly(ethylene glycol) (PEG) as a hydrophilic part, and poly(butyl methacrylate)^53^, poly(D,L-lactide)^54^, poly(ε-caprolactone) (PCL)^55,56^ and poly(propylene glycol)^57^, respectively, as a hydrophobic core. Slightly higher loading capacities (from 7 to 12%) were reported for PEG-PCL micelles loaded with Dex-acetate. However, these micelles exhibited limited stability in saline (<2 h)^58^.

The P1–P5 formulations have to be stable in the physiologically-relevant environment in order to be considered as potential drug nanocarriers. Instabilities can result in an uncontrolled aggregation and precipitation that would completely hamper obtaining reliable biological data. The stability and size of Dex-loaded micelles were determined by means of a dynamic light scattering (DLS) in PBS by monitoring the size of micelles for 24 hours. This time range was selected based on the work of He *et al*.^42^, where the authors described near quantitative (>80%) release of loaded drug from POx micelles within 24 h, albeit for different POx/drug system. This time range is also relevant for the presently studied system. Two drug concentrations were selected, namely 1 and 2 g∙L^−1^, to investigate the effect of loaded Dex on micellar stability. The concentration of 2 g∙L^−1^ corresponds to formulations affording the maximum loading capacity. The copolymer concentration was kept constant of 10 g∙L^−1^ for all formulations as previously used in drug formulation studies with POx micelles^41,42^.

**Figure 3:**
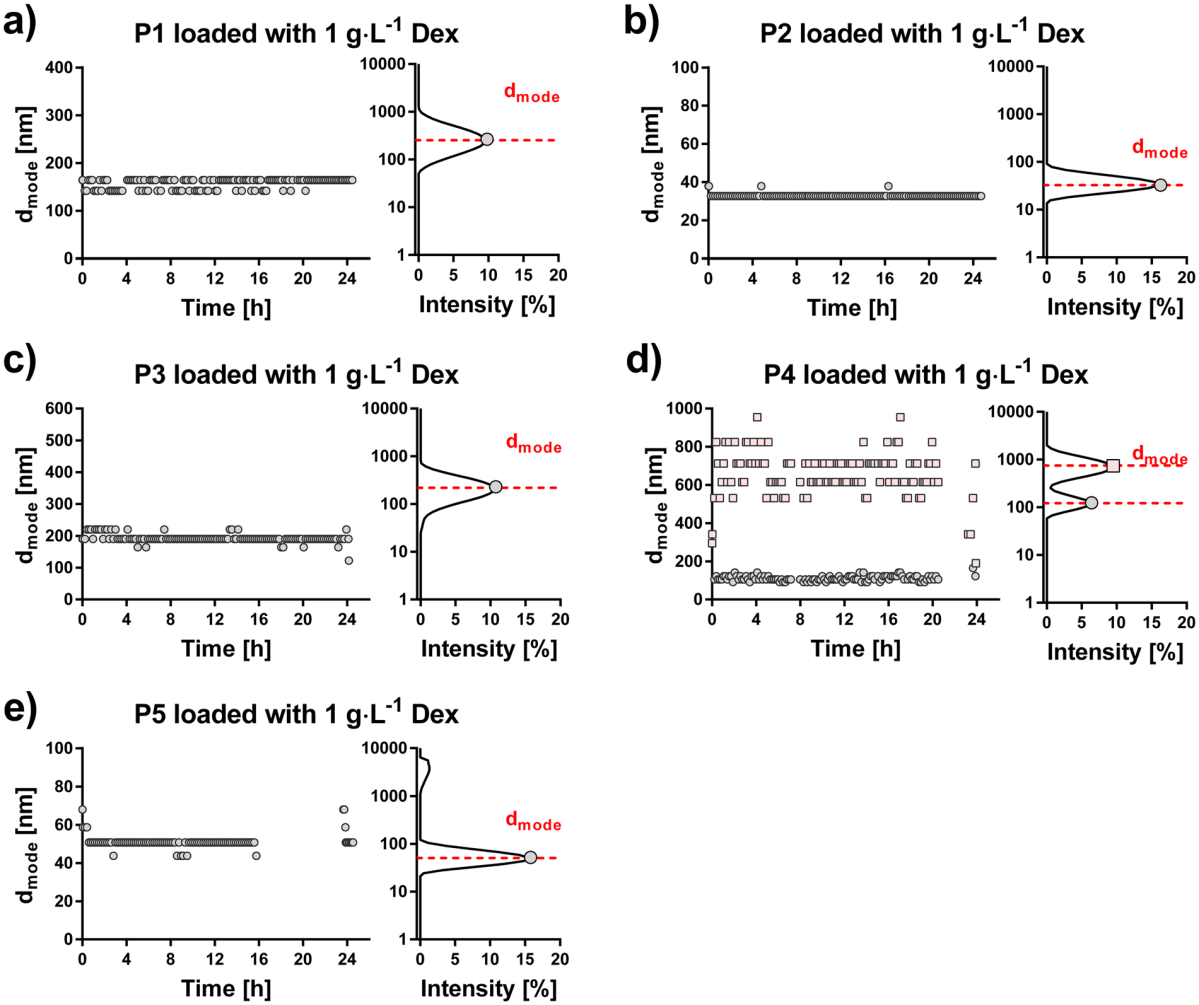
Stability test of micelles loaded with 1 g∙L^−1^ Dex. Dynamic light scattering measurements to assess micellar stability over 24 h in PBS. For each micelle type, the left plot represents the diameter of micelles at different time-points expressed as a peak maximum from intensity size distribution (*D*_mode_), and the right plot shows a representative intensity size distribution 12 h after the preparation. For micelles loaded with 2 g∙L^−1^ Dex refer to Supplementary Fig. S2.

The DLS data for micelles loaded with 1 g∙L^−1^ Dex are shown in Fig. 3 and in Supplementary Table S1. The micelle diameter is expressed as a peak maximum derived from intensity size distribution (*D*_mode_). For P1-P3 and P5 micellar systems, the intensity size distributions were monomodal and constant values during 24 h of analysis demonstrated their sufficient stability in PBS. During 24 h, the P4 system constantly exhibited a bimodal distribution with peak maxima at 113 nm and 667 nm, respectively. This copolymer also exhibited the lowest loading capacity (Fig. 2), which may be associated with a less stable formulation in this case. The DLS results concerning a 2 g∙L^−1^ Dex concentration are presented in Supplementary Table S1 and Supplementary Fig. S2. Overall, the micelles exhibited satisfactory stability although with a slight reduction in the 24 h observation, except for P2 sample. In some cases, a slight decrease in particle size was also observed (for copolymers P1, P3 and P4) and for P4 copolymer, the sample became polydisperse after 10 h.

**Figure 4:**
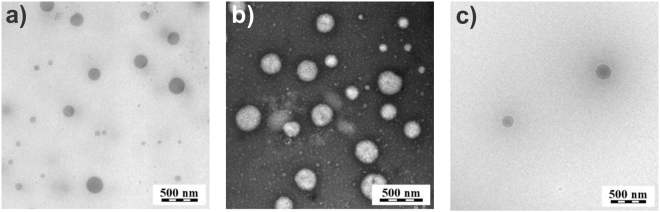
TEM micrographs of P1 micelles. Representative micrographs of P1 micelles (c_polymer_ = 10 g·L^−1^, c_dex_ = 1 g·L^−1^) measured by TEM without staining (**a**) with negative staining by uranyl acetate (**b**) and in cryo-TEM mode (**c**).

P2 and P5 Dex-loaded micelles exhibited diameters <100 nm. These sizes are in a typical range of other POx-based micelles^42^. Pluronic micelles typically display even lower diameter of around 10 nm^59^, which may be attributed, in some cases, to lower molar masses of Pluronic copolymers. Copolymers P1 and P3, featured by a higher hydrophilic fraction, formed micelles with diameters between 100 and 200 nm. These dimensions obviously exceed the typical polymeric micelles size, suggesting that these formulations may lead to polymersomes, worm-like micelles or micellar aggregates. Block copolymers with high hydrophilic fraction in the range of 80–90 mol% may form spherical aggregates composed of tens of micelles with the diameter above 100 nm^60^. Similar aggregates were observed by Hruby *et al*.^36^ and Trzebicka *et al*.^38^ for thermoresponsive POx copolymers. In general, the hydrophilic fraction of copolymers in the range from 10 to 40 mol% favors the formation of polymersomes or worm-like micelles^61^. Krumm *et al*.^62^ reported formation of polymersomes from triblock copolymers with 2-phenyl-2-oxazoline as a middle part, with the hydrophilic fraction of around 50 mol%. Our results suggest that POx copolymers with a lower molar hydrophilic fraction (P2 and P5) formed smaller and more stable micelles. The same trend was observed by Trzebicka *et al*.^38^ for diblock copolymers composed of 2-ethyl-2-oxazoline and 2-phenyl-2-oxazoline. On the other hand, our copolymers, which exhibit larger diameter (P1, P3 and P4), possess high hydrophilic fraction from 70 to 80 mol%. In view of previously published results^60–62^, for these copolymers we did not expect the formation of polymersomes, but rather micellar aggregates. To prove this assumption, we characterized the prepared nanoparticles by transmission electron microscopy (TEM). Representative TEM micrographs for P1 (c_polymer_ = 10 g·L^−1^, c_dex_ = 1 g·L^−1^) are shown in Fig. 4. TEM microscopy proved that the nanoparticles possessed a spherical shape, mostly without distinct core-shell contrast, and definitely not hollow spheres (polymersomes) and with an average size of ~160 nm. Representative TEM micrographs for the other sample types (c_polymer_ = 10 g·L^−1^, c_dex_ = 1 g·L^−1^) are shown in Supplementary Fig. S3. In agreement with DLS measurements, all samples contained particles with a rather broad size distribution. P2 and P5 samples exhibited particle sizes mostly below 100 nm. In sample P3, larger particles (around 200 nm) were observed. Sample P4 showed the largest particles with a size around 1 μm.

**Figure 5:**
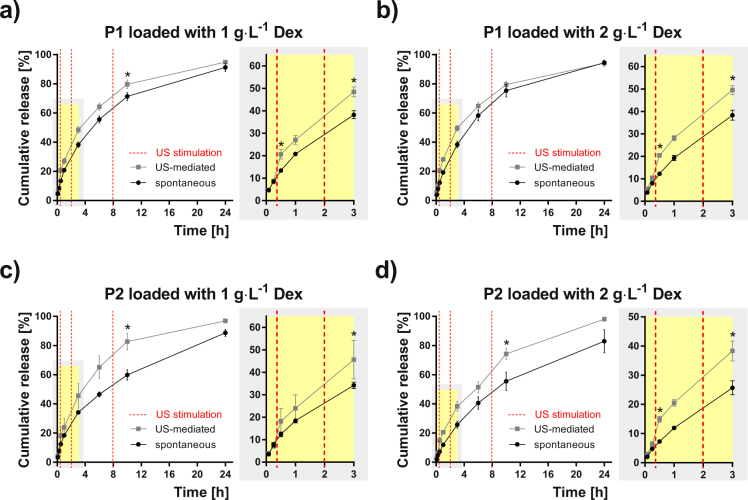
Dex release profiles for P1 and P2 Dex-loaded micelles. Spontaneous (black points) and US-mediated (grey points) for micelles based on POx diblock copolymers P1 (**a**,**b**) and P2 (**c**,**d**) loaded with 1 and 2 g∙L^−1^ Dex, respectively. The release data are expressed as the percentage cumulative Dex release with respect to the maximum release for a given micelle type. Each point shows the mean value and the standard deviation obtained from four measurements. Red dashed lines represent the time-points when the ultrasound stimulation was provided. The zoomed plots highlight the first 3 h of the release experiment. Asterisk indicates a statistically significant difference between US-mediated and spontaneous release values (p < 0.05, two sample Kolmogorov-Smirnov test).

**Figure 6:**
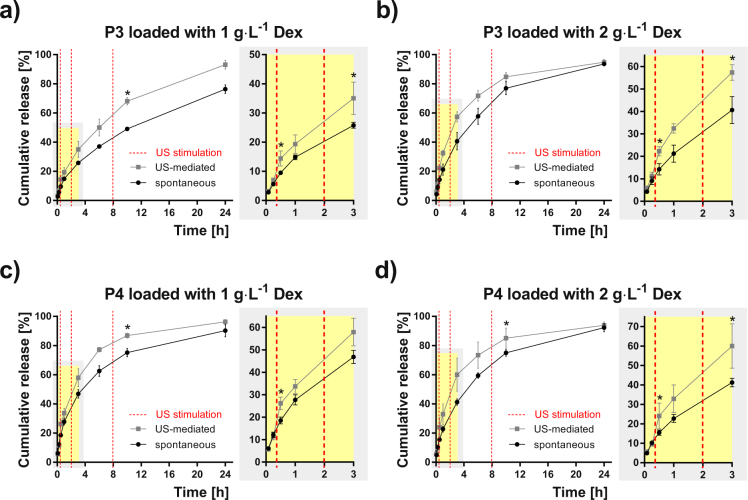
Dex release profiles from P3 and P4 micelles. Spontaneous (black points) and US-mediated (grey points) for micelles based on POx diblock terpolymers P3 (**a**,**b**) and P4 (**c**,**d**) loaded with 1 and 2 g·L^−1^ Dex, respectively. The release data are expressed as the percentage cumulative Dex release with respect to the maximum release for a given micelle type. Each point shows the mean value and the standard deviation obtained from four measurements. Red dashed lines represent the time-points when the ultrasound stimulation was provided. The zoomed plots highlight the first 3 h of the release experiment. Asterisk indicates a statistically significant difference between US-mediated and spontaneous release values (p < 0.05, two sample Kolmogorov-Smirnov test).

**Figure 7:**
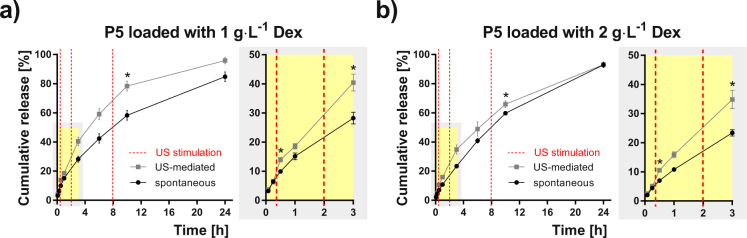
Dex release profiles from P5 micelles. Spontaneous (black points) and US-mediated (grey points) for micelles based on POx triblock copolymer P5 loaded with 1 and 2 g·L^−1^ Dex, respectively. The release data are expressed as the percentage cumulative Dex release with respect to the maximum release for a given micelle type. Each point shows the mean value and the standard deviation obtained from four measurements. Red dashed lines represent the time-points when the ultrasound stimulation was provided. The zoomed plots highlight the first 3 h of the release experiment. Asterisk indicates a statistically significant difference between US-mediated and spontaneous release values (p < 0.05, two sample Kolmogorov-Smirnov test).

We investigated US as a possible release-modulating stimulus for Dex release. We compared concentrations of 1 and 2 g·L^−1^ using P1–P5 micelles and spontaneous and US stimulated release (Fig. 1b). Supplementary Fig. S4 depicts the experimental protocol that is detailed in the Methods section. US stimulation (40 kHz, 20 W, 10 min duration) was provided with a US water bath at three time-points during the 24 h release experiment, specifically at *t*_1_ = 17 min, *t*_2_ = 2 h and *t*_3_ = 8 h. Figures 5–7 show the Dex release data obtained for all the micelle types and both Dex concentrations. The release profiles are reported as the cumulative percentage release at different time-points (5 min, 15 min, 30 min, 1 h, 3 h, 6 h, 10 h and 24 h). For all the formulations, there is a clear trend that the US stimulation increases the amount of released Dex compared to the spontaneous release. This is supported by statistically significant differences between respective data sets in most of the cases.

The effect of micelle hydrophilicity is highlighted in Supplementary Fig. S5 that compares the cumulative release data, depicted in Figs 5–7, for all the micelle types and both Dex concentrations. This comparison indicates a trend that, in case of spontaneous release, the micelles possessing a lower hydrophilic fraction (P2, P3, P5) exhibit slower release rates than micelles with a higher hydrophilic fraction. A slight effect of Dex concentration cannot be excluded since this trend is weaker for the concentration 2 g·L^−1^, due perhaps to the slight reduction in stability and size. Interestingly, the release profiles became similar after applying the US stimulation. This suggests that micelles of a less hydrophilic character are more sensitive to US than their counterparts of a more hydrophilic character. At the final time-point (24 h) of the release experiments, both US-stimulated and spontaneous release converged to similar release values in most cases. This is due to the relatively fast-releasing nature of this type of nanocarriers, which show almost complete release of the drug after 24 hours^42^.

Another option to capture the differences between US-mediated and spontaneous release is shown in Table 1. We quantified the increase of released Dex due to the US stimulation at a given time expressed as either the difference in release (Δ release in percentage) or as a slope difference (Δ slope in percentage) determined from the curves in Figs 5–7 at defined time intervals related to three US stimulations (detailed in the Methods section and Supplementary Fig. S6). The maximum Δ release (+105.4%) was found for P2 micelles loaded with 2 g∙L^−1^ Dex after the first US stimulation. The slope of the cumulative release generally increased for all the samples after the first and the second US stimulations showing positive Δ slope values. On the other hand, a reduction of the slope was observed after the third US stimulation (Δ slope negative values) for systems of a more hydrophilic nature. This can be attributed to a lower sensitivity of these formulations to the US-mediated release.

**Table 1:** Percentage difference of cumulative drug release and slope between spontaneous and US-stimulated release.

Sample		1^st^ US stimulation		2^nd^ US stimulation		3^rd^ US stimulation	
Micelle type	Dex concentration [g·L^−1^]	Δ Release [%]	Δ Slope [%]	Δ Release [%]	Δ Slope [%]	Δ Release [%]	Δ Slope [%]
P1	1	54.0	150.1	26.9	22.8	11.8	−1.8
	2	67.4	143.7	29.5	12.7	5.8	−13.8
P2	1	48.2	135.9	33.7	38.1	38.5	36.1
	2	105.4	243.0	50,1	31.5	35.2	58.0
P3	1	52.4	98.2	36.1	43.7	38.6	49.4
	2	60.5	123.1	43.8	29.8	10.5	−31.2
P4	1	42.0	129.1	24.0	27.0	15.7	−23.3
	2	56.1	183.3	45.8	46.5	13.5	−23.0
P5	1	41.0	117.0	43.7	67.1	34.8	22.0
	2	51.5	120.6	49.3	51.0	10.4	−9.5

Δ release values and Δ slope values were reported for the different time-points and time-intervals, respectively, for each US stimulation. Positive and negative Δ slope values indicate an increase and reduction, respectively, for the US-stimulated release compared to spontaneous release.

US can produce both mechanical and thermal effects in interaction with a target^63^. In our case, the temperature of the water bath was measured before and after the 10 min US stimulation in order to determine whether thermal effects contributed to the augmented release observed for the US-stimulated samples. The measured temperature increase was 1.4 ± 0.5 °C. Some of the copolymers comprise the thermoresponsive nPropOx blocks (P1, P2, P5). In view of our previously published data on similar systems^50^, we conclude that these copolymers of a given composition should not exhibit a relevant thermosensitive behavior in this temperature interval. In other words, the observed release data are not influenced by this minor temperature fluctuation during the US-mediated release experiment but they are a result of mechanical effects of US on micellar structure, i.e. transient cavitation, as proposed by Husseini *et al*.^19^ for the US-triggered release of doxorubicin from Pluronic P105 micelles. In our case, ultrasonic stimulation did not cause an irreversible degradation of the polymer chains. CryoSEM images (Supplementary Fig. S7) show in fact that polymeric micelles maintained a spherical shape both before and after ultrasonication. Therefore, enhanced drug delivery effects due to US are probably due to a reversible physical de-assembly of the micelles upon stimulation.

Additionally, the release profiles were fitted with mechanistic zero-order and empirical/semi-empirical Ritger-Peppas models^64^. The parameters found for both these models are reported in Supplementary Table S2. The best and worst fittings curves for the two models are reported in Supplementary Fig. S8. The zero-order model is appropriate for drug reservoir systems of constant properties in which the initial drug concentration exceeds the drug solubility. For this case, the fit was rather poor with a R^2^ = 0.89 obtained for the spontaneous release of P5 micelles with 2 g∙L^−1^ Dex. A much better fit was obtained using the Ritger-Peppas model. The release rate constants *K* were always higher for the case of US-mediated release profiles. The *n* exponents were in the range typical for a drug release mechanism of an anomalous transport, i.e. the combination of drug diffusion and polymer swelling and perturbation, which for a spherical drug carrier system is 0.43 and 0.85^64^. This is the case for both spontaneous and US-mediated drug release profiles.

In summary, we examined the possibility to modulate the release of Dex from POx-based micelles by US. This principle can be successfully applied in both short- and long-term drug delivery using micellar systems. In the former case, micelles travel quickly once administered systemically in the bloodstream with a circulation time in the range of minutes^65^. Thus, having an outer tool (an ultrasound probe) able to enhance the release kinetics in a localized body/tissue portion, even if for few minutes/hours, may have a high clinical relevance. This fully justifies the releasing nature of these nanocarriers, showing almost 100% release of the drug after 1 day, in some targeted drug delivery scenarios. We analyzed POx-based micelles loaded by dexamethasone, a glucocorticoid, that is used in the clinics as anti-inflammatory and immunosuppressive agent. The action of this drug can be relevant for several biomedical applications: dexamethasone-loaded micelles administered systemically in the bloodstream have demonstrated an effective antitumor activity but have been also used for the treatment of rheumatoid arthritis ocular diseases and other pathologies dealing with inflammatory states.

A technology allowing a precise modulation of Dex release, enhanced through the US waves, can be beneficial for long-term applications. This would enable to tune/minimize undesired body reactions, such as an excessive fibrotic response to neural interfaces^66^, implanted artificial organs^67^, biomaterials^68^ and other medical devices. In such systems, the POx-based micelles may be combined with additional materials reducing the release rates.

## Methods

### Synthesis of monomers

Monomers were synthesized by adapting known procedures^69,70^, as reported in detail in the Supplementary Materials (section S1). The NMR spectra of prepared compounds are depicted in Supplementary Figs S9–S11.

### Synthesis of copolymers

#### MetOx_n_-b-nPropOx_m_ (P1, P2)

Two diblock copolymers from 2-methyl-2-oxazoline and 2-*n*-propyl-2-oxazoline differing in chain length and ratio of blocks were prepared. The synthesis of P1 was performed as follows. The initiator methyl 4-nitrobenzenesulfonate (MeONs, 71.9 mg, 0.331 mmol) was dried in a Schlenk flask under reduced pressure for 1 h. Subsequently, benzonitrile (12 mL) and 2-methyl-2-oxazoline (4.7 g, 55.2 mmol) were injected into the flask. The polymerization of the first copolymer block proceeded under stirring in oil bath at 100 °C for 24 h. Then, 2-*n*-propyl-2-oxazoline (1.5 g, 13.3 mmol) and benzonitrile (10 mL) were added into the reaction mixture, which was polymerized at 100 °C for further 24 h until full conversion was achieved, as confirmed by ATR-FTIR measurement. Termination was performed by treatment of the cooled polymerization mixture with 1 M methanolic KOH (1 mL) for further 3 h. Resulting diblock copolymer was precipitated into cold diethylether (250 mL). Dried copolymer was dissolved in distilled water and purified by dialysis against distilled water (SpectraPor® of 1 kDa molecular weight cut-off, Spectrum Laboratories, Inc., USA) for 72 h and freeze-dried. Resulting diblock copolymer was achieved as white powder (yield P1 = 5.0 g, 81%).

Copolymer P2 was prepared accordingly, using 72.3 mg (0.333 mmol) of MeONs and 1.09 g (12.8 mmol) of 2-methyl-2-oxazoline in 4 mL of benzonitrile for the first step, and 1.9 g (16.8 mmol) of 2-n-propyl-2-oxazoline in 5.5 mL of benzonitrile for the second step. Following the same work-up procedure as for P1, the product was obtained as yellow powder with the yield 74% (2.16 g).

#### MetOx_m_-b-(ButOx_n1_-EnOx_n2_)_stat_ (P3, P4)

Diblock copolymers containing double bonds were prepared in two sequential steps starting from hydrophobic block based on 2-butyl-2-oxazoline (ButOx, 2) and 2-butenyl-2-oxazoline (EnOx, 3) in statistical arrangement. The preparation of block copolymer P3 with composition MetOx/ButOx/EnOx = 40/20/10 was performed as follows. The initiator MeONs, (0.134 g, 0.62 mmol) was dried in a Schlenk flask under reduced pressure for 1 h. Subsequently, acetonitrile (4 mL), ButOx (1.57 g, 12.34 mmol) and EnOx (0.77 g, 6.15 mmol) were injected into the flask and solution was degassed by three freeze-thaw cycles. The reaction mixture was stirred in oil bath at 80 °C for 24 h, until the full conversion was achieved, as confirmed by ATR-FTIR. Subsequently, MetOx (2 mL, 23.62 mmol) and acetonitrile (4 mL) were added into the reaction mixture, which was polymerized for further 24 h at the same temperature until full conversion was achieved. Termination was performed by treatment of the cooled polymerization mixture with methanolic KOH for further 3 h. Resulting diblock copolymer was precipitated into cold diethylether (200 mL). Dried copolymer was purified by dialysis against distilled water (SpectraPor® of 1 kDa molecular weight cut-off, Spectrum Laboratories, Inc., USA) for 72 h and freeze-dried. Resulting diblock copolymer was obtained as white powders (yields P3 = 2.3 g, 53%).

Copolymer P4 was prepared accordingly, starting with 53 mg of MeONs (0.244 mmol), 0.87 g of ButOx (6.84 mmol), 0.3 g of ButEnOx (2.4 mmol) in 2.4 mL of acetonitrile as a first step. As a second step, 2 g of MetOx (23.5 mmol) in 4 mL of acetonitrile was added. Following the same work-up procedure as P3, the product was obtained as white powder (yield 1.45 g, 46%).

#### MetOx_25_-b-nPropOx_145_-b-MetOx_25_ (P5)

Triblock copolymer consisting of 2-methyl-2-oxazoline and 2-*n*-propyl-2-oxazoline was synthesized as previously described^50^. To a pre-dried Schlenk flask initiator 1,3-propanediol di-*p*-tosylate (193 mg, 0.5 mmol), dissolved in benzonitrile (25 mL), was added. Subsequently, 2-n-propyl-2-oxazoline (8.21 g, 72.6 mmol) was added. The reaction mixture was stirred in an oil bath at 100 °C until the full conversion was achieved as verified by ^1^H-NMR. The polymerization mixture was then cooled with an ice bath and 2-methyl-2-oxazoline (2.13 g, 25.0 mmol) and benzonitrile (8 mL) were added under inert atmosphere. The mixture was then stirred at 100 °C until the full conversion of the second monomer was achieved. The polymerization was terminated by adding an excess of piperidine (0.76 g). After several hours of stirring at room temperature (RT), an excess of K_2_CO_3_ was added. The product was purified by repeated precipitation in cold diethyl ether (350 mL) followed by dialysis against distilled water (MWCO: 1 kDa; Spectrum Laboratories, Inc., USA) and freeze-dried. The polymer was obtained as a white powder (yield 45%).

The NMR spectra and polymerization reaction schemes are depicted in Supplementary Figs S12–S19.

### Analytical methods

#### ^1^H NMR

^1^H NMR spectra of all compounds were recorded at room temperature on a Varian VXR-400 (Varian, USA) in CDCl_3_ and methanol-d_4_ solutions using tetramethylsilane (TMS) as an internal standard.

### Size exclusion chromatography

The size exclusion chromatography (SEC) characterization was performed using a P102 pump (Watrex, Czech Republic) and an evaporative light scattering detector ELS–1000 (PL-Agilent Technologies, Stretton, UK) with the temperature set to 180 °C and the gas flow rate of 1.5 mL.min^−1^. The TSK gel GMH hr - M, 300 × 7.5 mm (TosoHaas Bioscience) SEC column was used for separation. The SEC analysis was performed at ambient temperature. The mixture of 50 wt.% *N*,*N*-dimethylformamide (HPLC grade 99.7%, Alfa Aesar) and 50 wt.% chloroform (HPLC grade 99.8%, Sigma-Aldrich) was used as SEC eluent at the flow rate of 1 mL.min^−1^ following the conditions used in Zahoranova *et al*.^50^. The calibration was based on narrow polystyrene standards (580–100 000 g∙mol^−1^, Pressure Chemical Company, Pittsburgh, US) providing the molar mass data for synthesized copolymers relative to polystyrene standards. Data were collected and processed using the Clarity software (DataApex, Czech Republic).

### ATR-FTIR

The ATR-FTIR spectra were recorded with Infrared spectrophotometer NICOLET 8700TM (Thermo Scientific, USA), with 64 scans and resolution of 4 cm^−1^.

### Preparation of Dex-loaded micelles

POx-based micelles were prepared according to the thin film hydration method described elsewhere^42^. Stock solutions of copolymers and Dex in ethanol were prepared (usually 100 g∙L^−1^ and 10 g∙L^−1^, respectively). The stock solutions were then pipetted into glass vials to obtain desired ratios of polymer to Dex. Ethanol was subsequently evaporated using a heat gun to obtain a thin film on the glass surface. The vials were dried at 60 °C in vacuum oven. Prior to experiment, polymer layers were re-hydrated using appropriate amounts of distilled water or PBS (200 μL for HPLC measurements, 1 mL for DLS measurements, 100 μL for drug release study). The resulting solutions were resuspended by Vortex, centrifuged (3,000 rpm for 10 min) in order to remove non-loaded Dex in a form of precipitated flocks, and the supernatant was used in further experiments immediately after the preparation.

### High performance liquid chromatography

The amount of Dex loaded in micelles was measured using Agilent 1200 Series HPLC System (UV detection at 240 nm, Zorbax Eclipse Plus column C18, 4.6 ∗ 250 mm, 5 µm, eluent methanol/water 90/10). For these measurements, the layer containing a copolymer and Dex was re-hydrated by adding of 200 µL of distilled water to obtain final concentration 10 g∙L^−1^ of a copolymer and 0.5–10 g∙L^−1^ of Dex. The solution was further centrifuged (3,000 rpm for 10 min) in order to remove non-loaded Dex, 100 µL of the sample were mixed with 900 µL of methanol and measured by HPLC.

The loading efficiency was calculated as the weight percentage of loaded Dex, *m*_Dex_, from the original Dex feed, *m*_Dex,0_: *LE* = (*m*_Dex_/*m*_Dex,0_) ∗ 100. The loading capacity was calculated as the percentage of loaded Dex with respect to the total weight of the formulation: *LC* = (*m*_Dex_/(*m*_Dex_ + *m*_copolymer_)) ∗ 100, where *m*_copolymer_ is the weight of copolymer in the formulation. The amounts of loaded Dex were calculated from calibration against free Dex dissolved in HPLC eluent (methanol:water 90:10) in the concentration range from 0.01 to 0.1 g∙L^−1^. The experiment was performed in quadruplicates.

### Dynamic light scattering

Dynamic light scattering (DLS) measurements were performed using Zetasizer Nano-ZS (Malvern Instruments, UK) equipped with a 4 mW helium/neon laser (λ = 633 nm) and thermo-electric temperature controller, with the following parameters set for poly(2-ethyl-2-oxazoline): refractive index = 1.520, absorbance = 0.001. All measurements were performed at 28 °C in measurement angle 173°. The micelles for this analysis were prepared to obtain final concentration 10 g∙L^−1^ of copolymer and 1 g∙L^−1^ or 2 g∙L^−1^ of Dex in PBS. The diameter (*D*_mode_) of the polymeric micelles was expressed as the peak maximum from the intensity size distribution. The stability measurements of the micelles were performed immediately after the preparation in the time interval 24 h. Typically, 200–400 measurements were done for each sample and averaged value of the diameter (*D*_mode_) from these measurements was compared (see Supplementary Table S1).

### Transmission electron microscopy

TEM and cryo-TEM observations were performed through a Tecnai G2 Spirit Twin 12 (FEI, Czech Republic), equipped with cryo-attachment (Gatan, cryo-specimen holder) using a bright field imaging mode at an accelerating voltage of 120 kV. For TEM measurements, sample solutions (c_polymer_ = 10 g·L^−1^, c_dex_ = 1 g·L^−1^) were dropped onto a copper TEM grid (300 mesh) coated with thin, electron-transparent carbon film. After 1 min the solution was removed by touching the grid bottom with filtering paper (fast drying method). Such a fast removal of the solution was performed in order to minimize oversaturation during the drying process. Before observing them, samples were left to dry completely at room temperature.

In order to confirm the observed nanoparticle morphology, one selected sample (P1) was visualized by two alternative TEM techniques: (i) TEM after negative staining with uranyl acetate solution (2 wt. %) as described elsewhere^71^ and (ii) cryo-TEM as described in the following paragraph.

For CryoTEM measurement, 3 μL of the sample solution were deposited on an electron microscopy grid covered with lacey carbon supporting film (Agar Scientific) after hydrophilization by glow discharge (Expanded Plasma Cleaner, Harrick Plasma, USA). The solution excess was removed by blotting (Whatman no. 1 filter paper) for ∼1 s, then the grid was immediately plunged into liquid ethane held at −181 °C. The frozen sample was immediately transferred into the microscope and observed at −173 °C under the conditions described above.

### Dex release study

Immediately after micelles preparation, the supernatants containing micelles (100 μL) were inserted in dialysis devices (Slide A -Lyzer® MINI Dialysis Units, 3,500 MWCO - Thermo Fisher Scientific). The devices were then placed in centrifuge tubes (2 mL volume) containing PBS at the initial time point (See Supplementary Fig. S4 for the drug release protocols schemes). For spontaneous release protocol, the dialysis device was moved from one centrifuge tube to another at each time point (5 min, 15 min, 30 min, 1 h, 3 h, 6 h, 10 h and 24 h). The Dex release from micelles was measured through absorbance readings (Lambda 45 UV/VIS Spectrometer, PerkinElmer, λ = 243 nm) of the PBS contained in each centrifuge tube (1.5 mL). After 24 h the micelles were exposed to 2 h sonication provided with the ultrasound water bath and absorbance measures were performed in order to evaluate the maximum Dex release (considered as 100%). Four different samples were analyzed for each micelle type (P1–P5) and for each of the Dex concentrations (1 and 2 g∙L^−1^). The US-mediated protocol was the same of the spontaneous one except for US stimulation, which was provided to the micelles over 10 min with a water bath, by setting it at a 20 W power and a 40 kHz frequency, for three time-points (17 min, 2 h and 8 h). Also in this case four different samples were analyzed for each micelle type (P1–P5) and for each Dex concentration (1 and 2 g∙L^−1^).

Both the US mediated and the spontaneous Dex release profiles are reported as the percentage cumulative Dex release respect to the maximum release in Figs 5–7. Differences between the spontaneous and the US-triggered drug release extent were evaluated at the time points after US stimulation and reported as respective increases, Δ Release and Δ Slope, for each copolymer and each Dex concentration (see Table 1 and Supplementary Fig. S6). Δ Release was calculated at 3 different time points (30 min, 3 h and 10 h) as the percentage difference of the cumulative release caused by US: *Δ Release (t*) = *((US*_*PCR*_ *(t*) − *S*_*PCR*_ *(t))/S*_*PCR*_* (t)) ∗ 100*, where *US*_*PCR*_ *(t)* and *S*_*PCR*_ *(t)* are US mediated and spontaneous release, respectively, at the time *t* after US stimulation. Δ Slope was calculated for 3 different time intervals (from 15 min to 30 min, from 1 h to 3 h, and from 6 h to 10 h) as the percentage variation of the slope of the release profiles caused by US: *Δ Slope (t*_*1–2*_*)* = *((US*_*slope*_ *(t*_*1–2*_*)* − *S*_*slope*_ *(t*_*1–2*_*))/S*_*slope*_ *(t*_*1–2*_*)) ∗ 100*, where *US*_*slope*_ *(t*_*1–2*_*)* and *S*_*slope*_ *(t*_*1–2*_*)* are the slopes of US mediated and spontaneouse release profiles, respectively, in the US stimulation containing time interval *t*_*1–2*_. Δ Release and Δ Slope computation is depicted in Supplementary Fig. S6. Data of the percentage cumulative release profiles were fitted with the zero-order model and with Ritger-Peppas model by means of a MATLAB^®^ toolbox (cftool) for curve fitting. The parameters are reported in Supplementary Table S2.

### Statistical Analysis

The percentage cumulative release values from US-mediated and spontaneous release experiments were compared at the time points immediately after the US stimulation (30 min, 3 h and 10 h) by using a non-parametric statistical analysis: a two-sample Kolmogorov-Smirnov test was performed in order to evaluate significant differences. Results were considered statistically different for p-values ≤ 0.05.

## Electronic supplementary material

Supplementary Information
