# Supplementary material for: Investigation of drug release modulation from poly(2-oxazoline) micelles through ultrasound
Source: Sci Rep. 2018 Jul 2;8:9893. doi: 10.1038/s41598-018-28140-3 (PMC6028437; doi:10.1038/s41598-018-28140-3)

Supplementary information

Investigation of drug release modulation from poly(2-oxazoline) micelles through ultrasound

Alice Rita Salgarella,[a,#] Anna Zahoranová,[b,#] Petra Šrámková,[b] Monika Majerčíková,[b,c] Ewa Pavlova,[d] Robert Luxenhofer,[e] Juraj Kronek,[b] Igor Lacík[b] and Leonardo Ricotti[a*]

[a]*The BioRobotics Institute, Scuola Superiore Sant'Anna, Viale R. Piaggio 34, 56025 Pontedera (PI), Italy*

[b]*Department for Biomaterials Research, Polymer Institute of the Slovak Academy of Sciences, Dúbravská cesta 9, 845 41 Bratislava, Slovakia*

[c]*Institute of Natural and Synthetic Polymers, Faculty of Chemical and Food Technology, Slovak University of Technology, Radlinského 9, 812 37 Bratislava, Slovakia*

[d]*Institute of Macromolecular Chemistry, Academy of Sciences of the Czech Republic, Heyrovského nám. 2, 162 06 Prague 6, Czech Republic*

[e]*Functional Polymer Materials, Chair for Chemical Technology of Materials Synthesis, University of Würzburg, Röntgenring 11, 97070 Würzburg, Germany*

[#] *= equally contributed*

[*] *= corresponding author (leonardo.ricotti@santannapisa.it)*

Table S1. Analytical data of block copolymers. Theoretical number average molar mass ($M_{n,theor}$) and theoretical molar hydrophilic fraction (F_{theor}) calculated from the feed, $M_{n,exp}$ and dispersity ($Đ$) measured by SEC, hydrophilic molar fraction (F_{exp}) calculated from ^{1}H NMR spectra, $D_{mode,1}$, $D_{mode,2}$ diameters calculated from DLS measurements in PBS, for dexamethasone concentrations 1 and 2 g·L^{-1}, respectively (mean ± SD from 200 - 700 measurements performed from preparation up to 24 hrs).

**Sample type**	**Theoretical composition**	$M_{n,theor}$ **[kg.mol^{-1}]**	$M_{n,exp}$ **[kg.mol^{-1}]**	$Đ$	F_{theor} **[%]**	F_{exp} **[%]**	**Yield [%]**	$D_{mode,1}$ **[nm]**	$D_{mode,2}$ **[nm]**
**P1**	$MetOx_{160}$-b-$nPropOx_{40}$	18.2	13.1	1.44	80	82	81	158 ± 11	129 ± 28
**P2**	$MetOx_{40}$-b-$nPropOx_{60}$	10.2	11.5	1.27	40	51	74	33 ± 1	33 ± 0.0
**P3**	$MetOx_{40}$-b-($ButOx_{20}$-$EnOx_{10}$)$_{stat}$	7.2	7.8	1.22	57	68	53	193 ± 15	134 ± 34
**P4**	$MetOx_{100}$-b-($ButOx_{30}$-$EnOx_{10}$)$_{stat}$	13.6	10.5	1.32	71	79	46	667 ± 122, 113 ± 51	372 ± 178, 115 ± 30
**P5**	$MetOx_{25}$-b-$nPropOx_{145}$-b-$MetOx_{25}$	20.9	9.9	1.33	26	43	45	51 ± 3	55 ± 13

Table S2. Drug release model parameters for spontaneous and US-mediated release. Drug release profiles were fitted with two different drug release models: the zero-order and the Ritger-Peppas models. The parameters obtained are reported in the table as well as the R^2 values, representing the quality of the fitting.

Sample		Release	Zero-order		Ritger-Peppas		
**Micelle type**	**Dex concentration [g·L^{-1}]**		**K (h^{-1}) * 10-3**	**R^2**	**K (h^{-1})**	**n**	**R^2**
P1	1	spontaneous	3.2	0.6030	0.20220	0.5838	0.9992
		US mediated	3.3	0.4108	0.26100	0.5759	0.9804
	2	spontaneous	2.7	0.6341	0.19160	0.6321	1.0000
		US mediated	3.0	0.3643	0.27110	0.5592	0.9899
P2	1	spontaneous	3.4	0.7192	0.18040	0.5865	0.9986
		US mediated	4.0	0.5088	0.23520	0.6122	0.9845
	2	spontaneous	4.9	0.8326	0.11940	0.6969	0.9999
		US mediated	5.4	0.6971	0.19740	0.6150	0.9862
P3	1	spontaneous	2.6	0.7918	0.13910	0.5725	0.9950
		US mediated	3.1	0.7064	0.18780	0.5782	0.9875
	2	spontaneous	2.1	0.5953	0.21120	0.5974	0.9997
		US mediated	2.2	0.1964	0.30740	0.5799	0.9912
P4	1	spontaneous	2.1	0.3431	0.26490	0.5283	0.9961
		US mediated	2.2	0.1354	0.32340	0.5461	0.9775
	2	spontaneous	1.8	0.5258	0.22430	0.5575	0.9994
		US mediated	2.1	0.1486	0.31430	0.6006	0.9828
P5	1	spontaneous	3.4	0.7838	0.14880	0.5860	0.9997
		US mediated	4.0	0.6234	0.19360	0.6685	0.9936
	2	spontaneous	5.2	0.8921	0.11050	0.6797	0.9997
		US mediated	5.2	0.7617	0.15920	0.7141	0.9976

Figure S1. Loading capacity of different micellar formulations. Maximal loading capacity for POx-based **P1-P5** formulations compared to the loading capacity of various Dex-loaded micelles reported in the literature: a = Janas et al.,[1] b = Yang et al.,[2] c = Wang et al.,[3] and d = Nidhi et al.[4] Loading capacity is calculated as 100 x $m_{Dex}/(m_{Dex}+m_{copolymer})$.

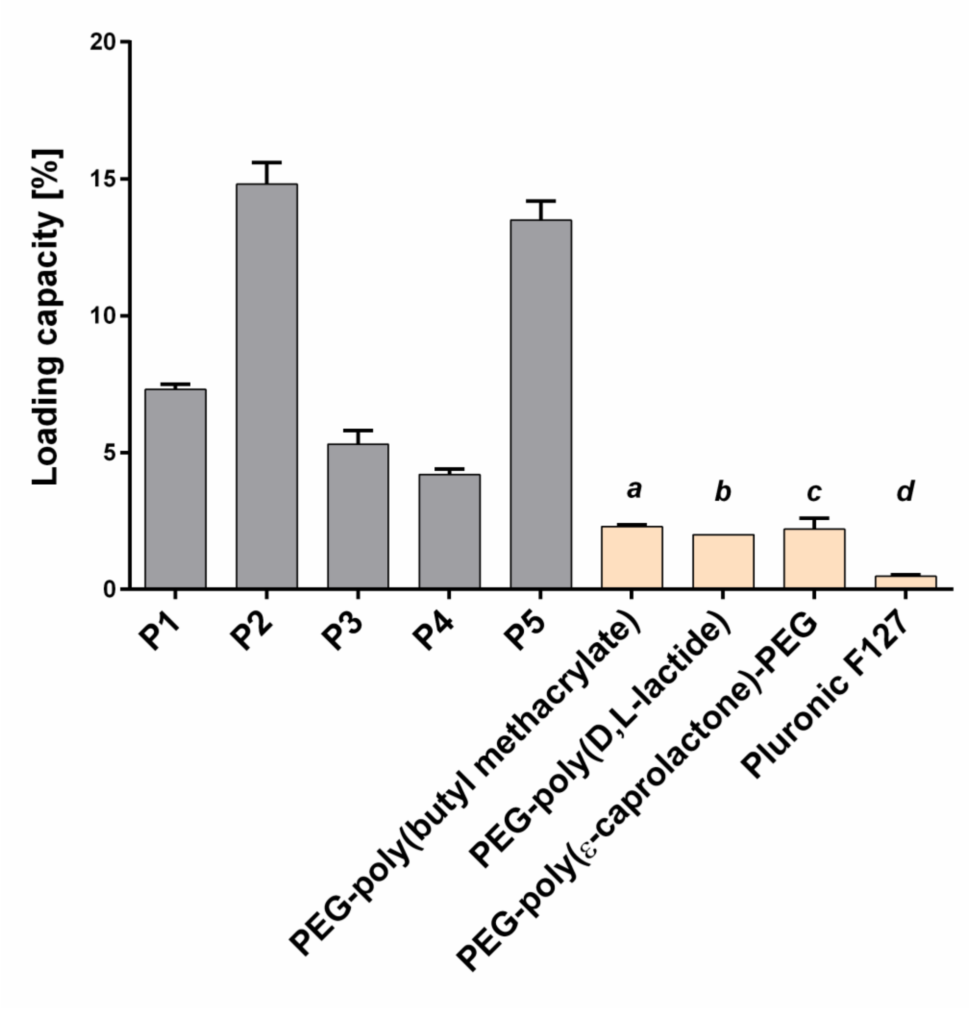

Figure S2. Stability test of micelles loaded with 2 g·L^{-1} Dex. Dynamic light scattering measurements to assess micelle stability over 24 h in PBS. For each micelle type, the left plot represents the diameter of micelles at different time-points expressed as a peak maximum from intensity size distribution (D_{mode}), and the right plot shows a representative intensity size distribution 3 hrs after the preparation. P4 Dex-loaded micelles exhibiting bimodal size distribution for the first 10 hrs (black line), then the sample became polydisperse and unstable (green line).

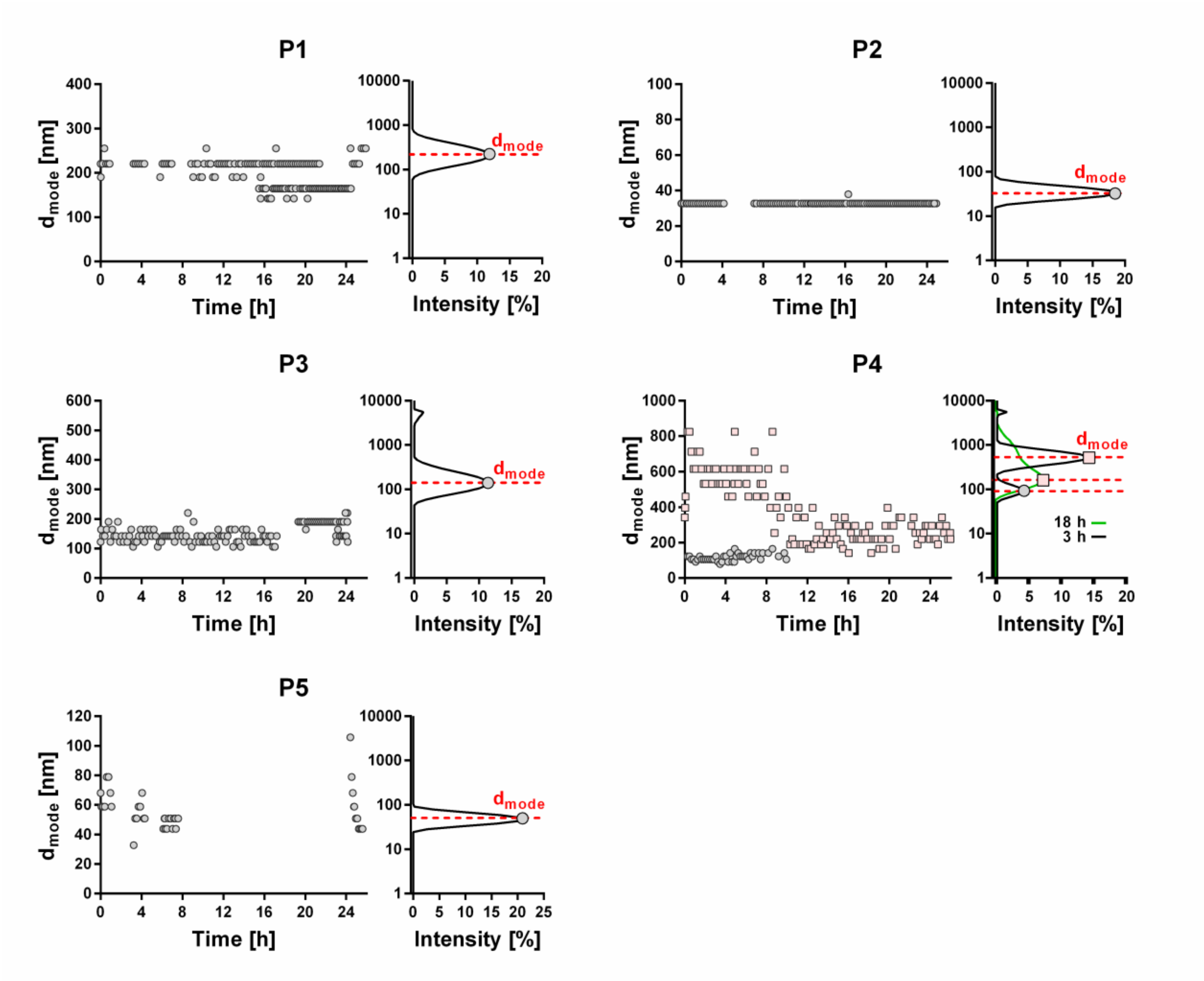

Figure S3. TEM micrographs of samples P2 - P5. Representative micrographs of samples P2 - P5 ($c_{polymer}$ = 10 g·L^{-1} , c_{dex} = 1 g·L^{-1}) measured using TEM. The samples were prepared by fast drying method.

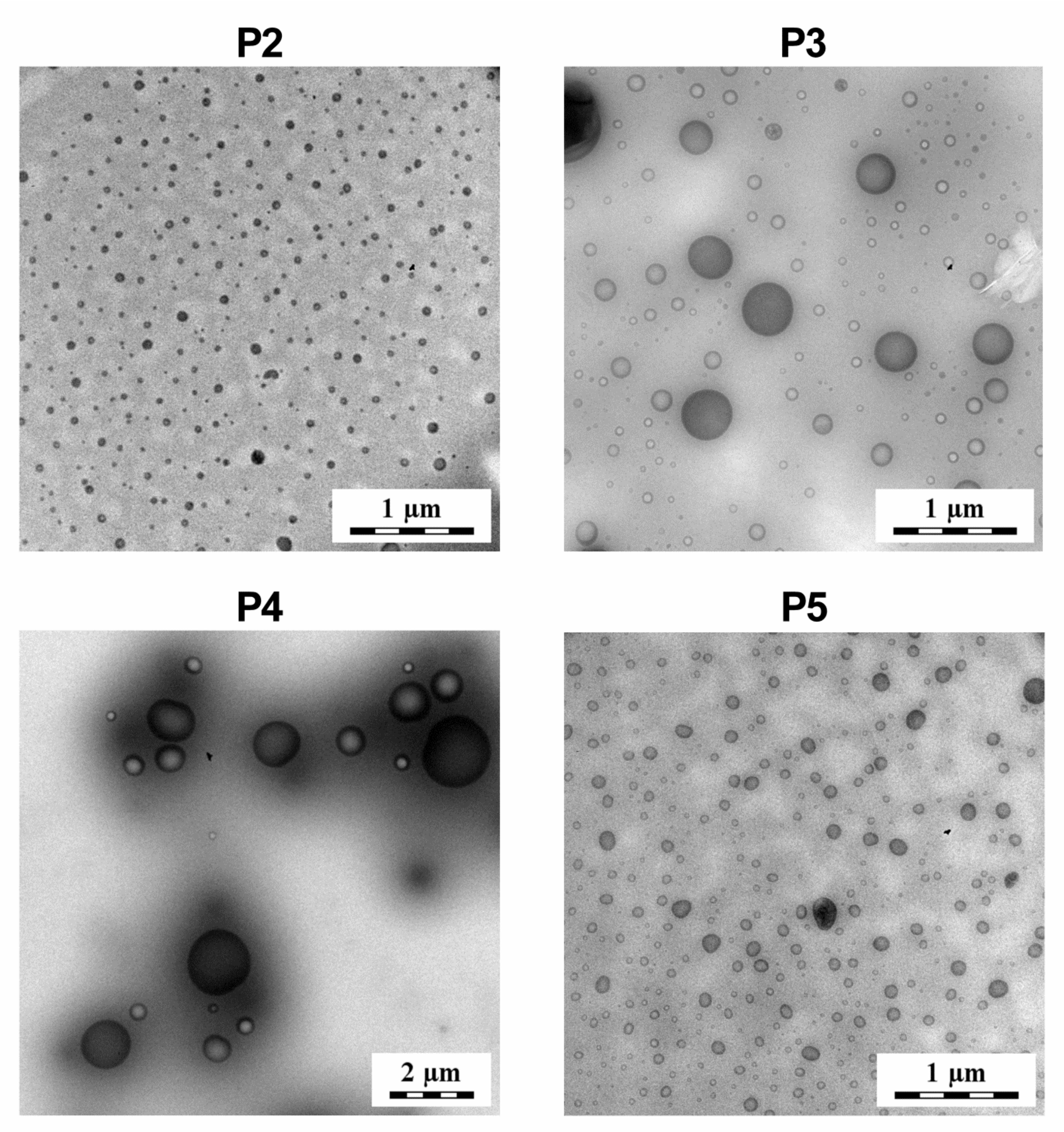

Figure S4. Drug release protocols. Poly(2-oxazoline) micelles were prepared by the thin film hydration method and then centrifuged for 10 min at 3,000 rpm. For each formulation, the supernatant containing micelles was inserted in a dialysis device (Slide-A-Lyzer® MINI Dialysis Units, 3,500 MWCO, Thermo Fisher Scientific) that was inserted in a centrifuge tube containing PBS. This represented the initial time point. For spontaneous release experiments, the dialysis device was moved from one centrifuge tube to another at each time point (5 min, 15 min, 30 min, 1 h, 3 h, 6 h, 10 h and 24 h). After 24 h the micelles were exposed to 2 h sonication provided with the ultrasound water bath (20 W, 40 kHz) and UV-vis measurements were performed in order to evaluate the maximum Dex release (considered as 100%). The US-mediated experiment was the same as for the spontaneous one except for US stimulation (20 W, 40 kHz), which was applied for 10 min at three time-points (17 min, 2 h and 8 h). Four different samples were prepared and analyzed for each micelle type (P1-P5) and for each Dex concentration (1 and 2 $g \cdot L^{-1}$).

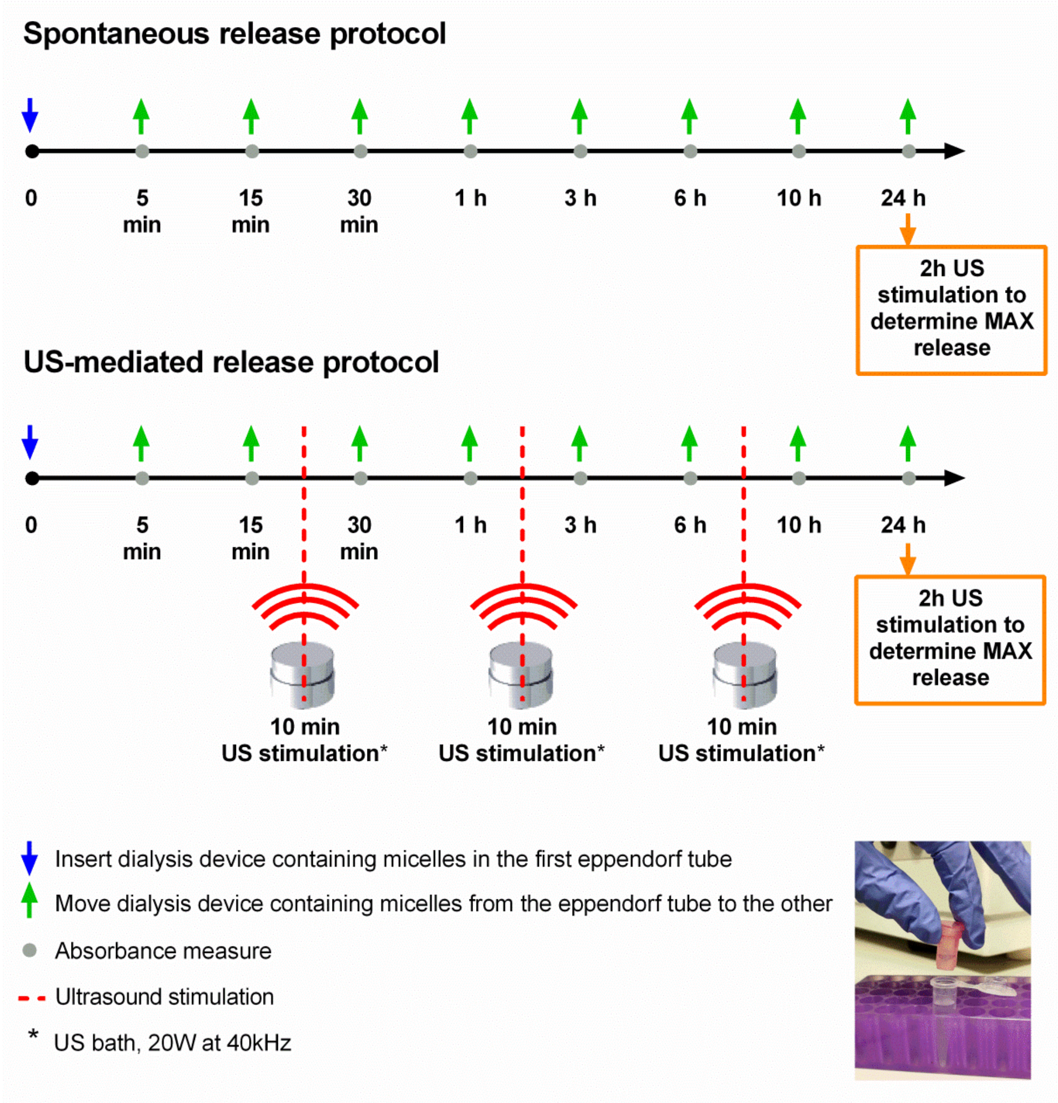

Figure S5. Comparison of cumulative release profiles with respect to copolymer hydrophilicities. The percentage cumulative release (mean values) of micelles possessing a higher hydrophilic fraction is depicted in blue color while the one of micelles possessing a lower hydrophilic fraction is depicted in red / orange color. a) shows the case of spontaneous and US-mediated release from micelles loaded with 1 g·L^{-1}concentration of Dex, while b) shows the profiles for 2 g·L^{-1} Dex concentration, without US stimulations d) Dex **c**= 2 g·L^{-1}, with US stimulations Note that cumulative release profiles are the same as in Fig. 5-7**.**

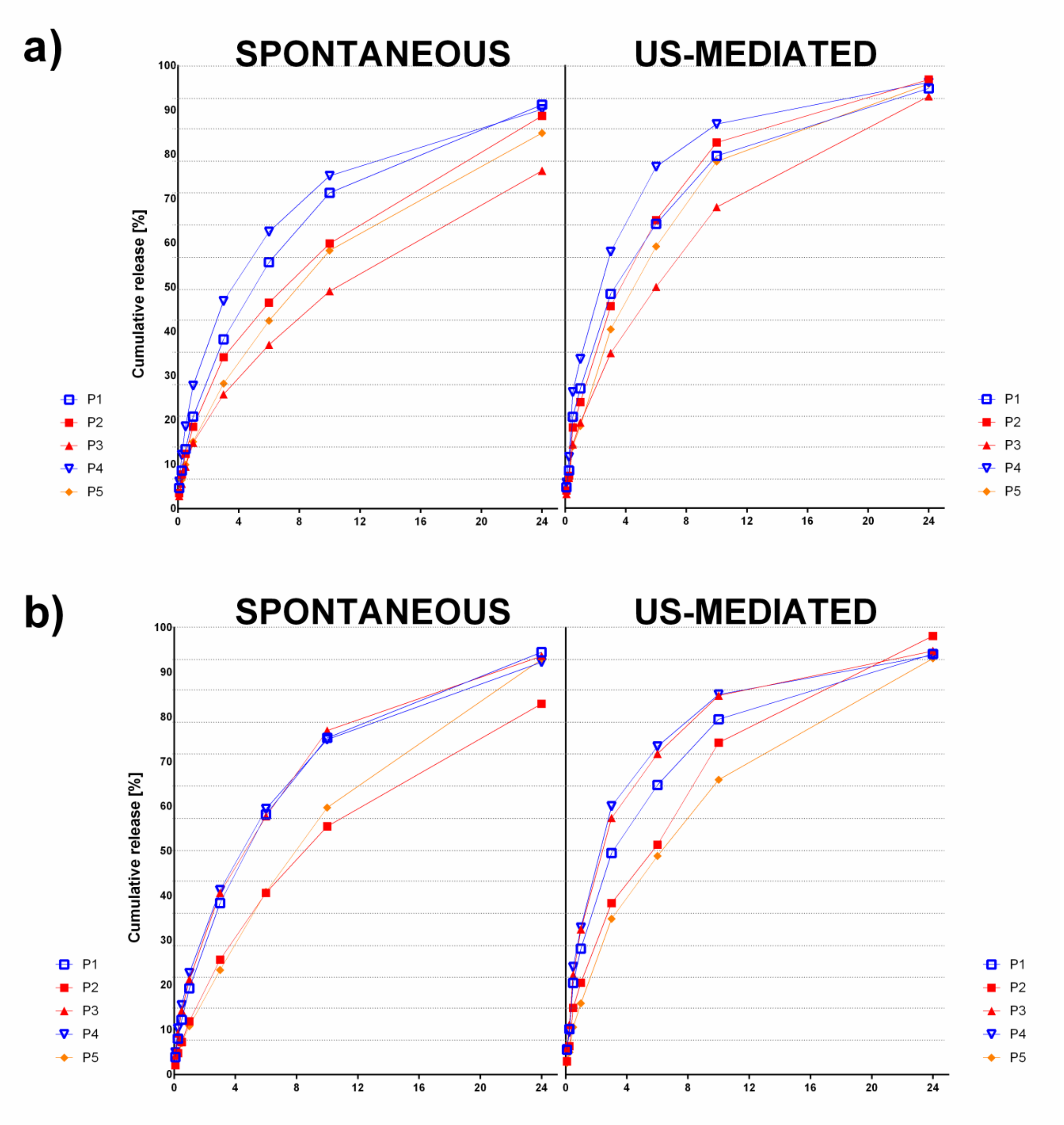

Figure S6. Δ Release and Δ Slope computation.

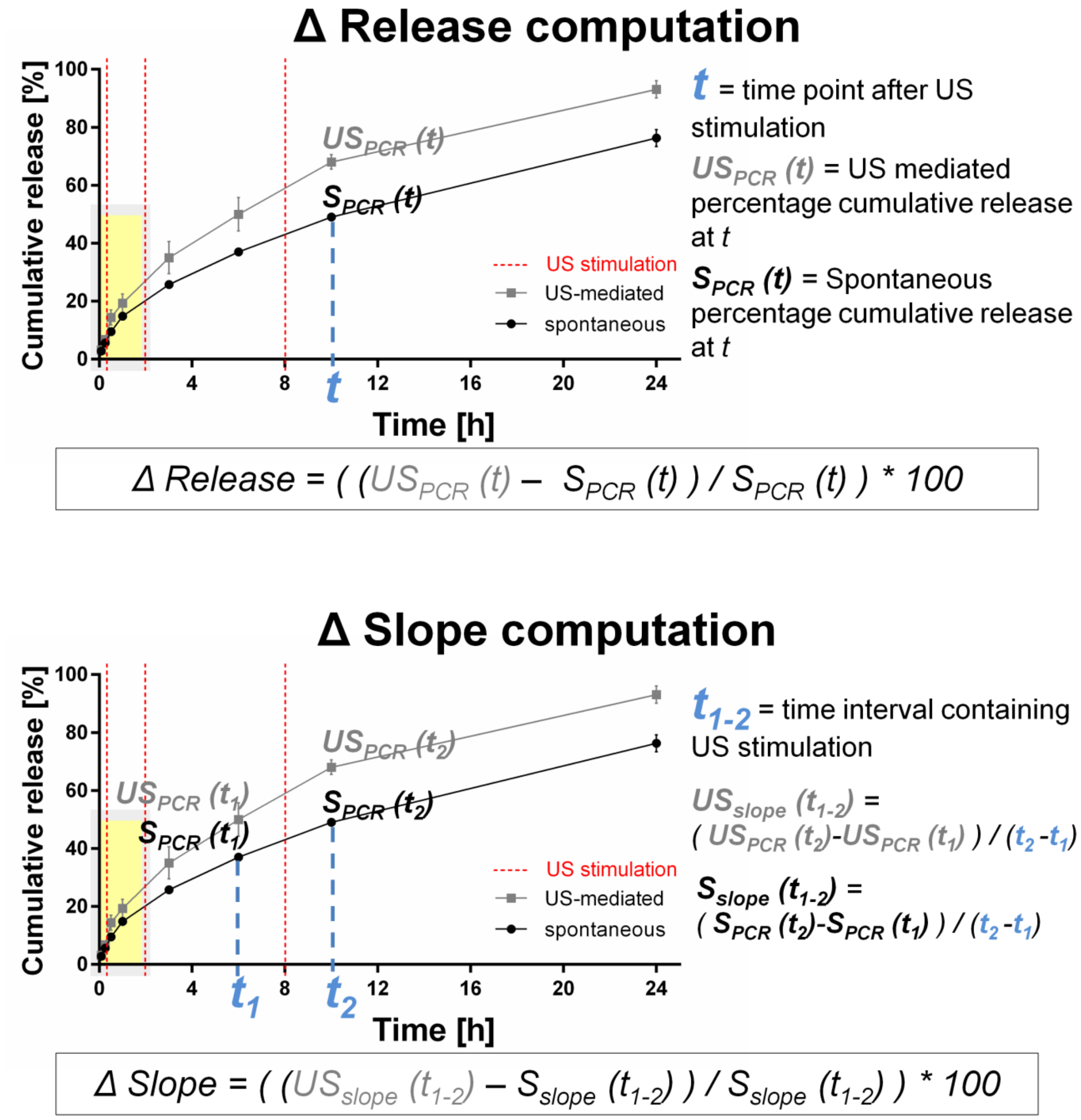

Figure S7. Representative SEC elugrams and CryoSEM images of copolymer P2 with and without the application of US. Representative SEC elugrams (a) of copolymer P2 with (red line) and without (black line) 30 min ultrasonication. The copolymer was in micellar form and provided with dexamethasone in water ($c_{polymer} = 10$ g·L^{-1}, $c_{dex} = 1$ g·L^{-1}). Elugrams show no evidence of degradation of polymer chains. CryoSEM (JEOL 7500 F, JEOL, Japan) equipped with cryo-mode system (Quorum, UK) images of P2 copolymer before (b) and immediately after (c) 10 min ultrasonication. The micelles exhibit spherical shape after ultrasonication, suggesting that the ultrasound-induced changes in the micelle structure are reversible.

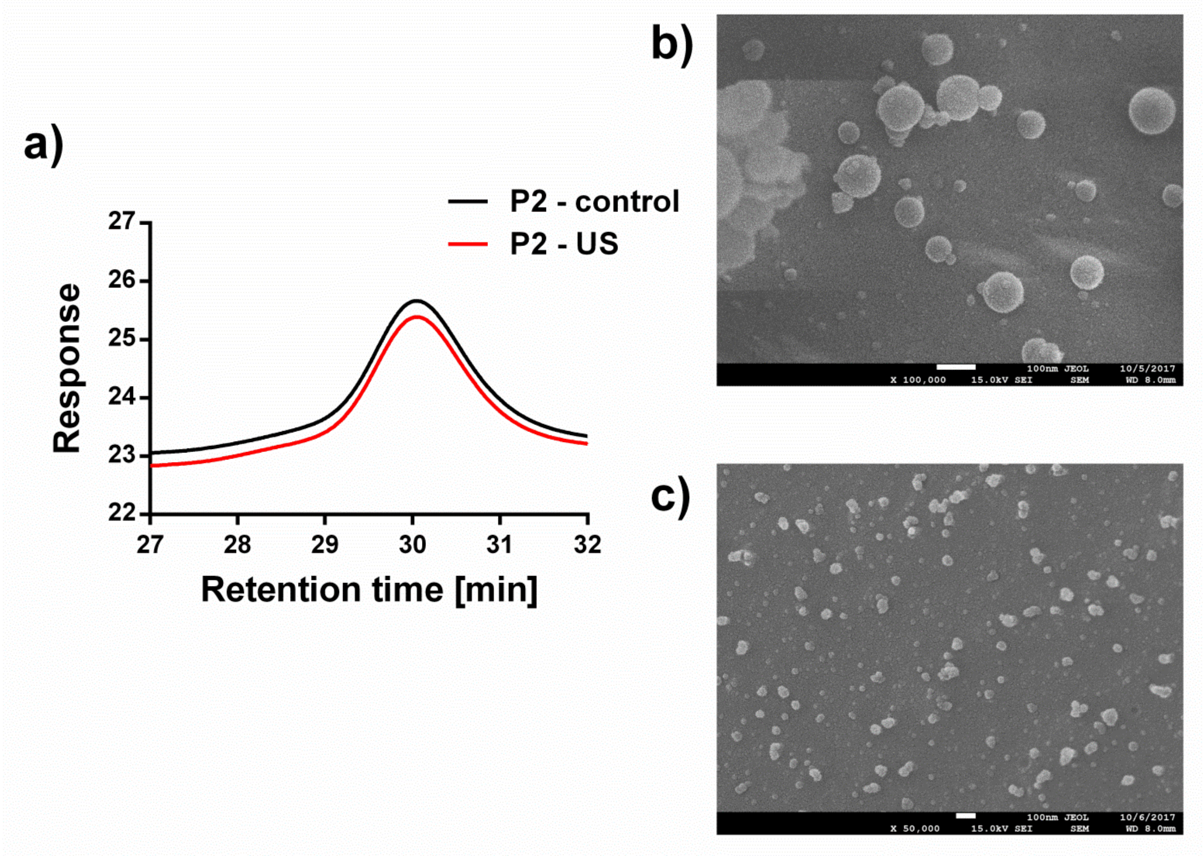

Figure S8. Representative model fitting curves. Representative fitting curves obtained by modeling the experimental Dex release data with two drug release models, namely zero-order and Ritger-Peppas model. For the two models, the sample fitting resulting in the highest R2 values (left graphs) and the ones featured by the lowest R2 values (right graphs) are reported. It is worth mentioning that, according to the Ritger-Peppas model assumptions, only the first five time points are represented, since they show a fraction of released Dex which is lower than 0.6 (consequently, a percentage cumulative Dex release smaller than 60%).

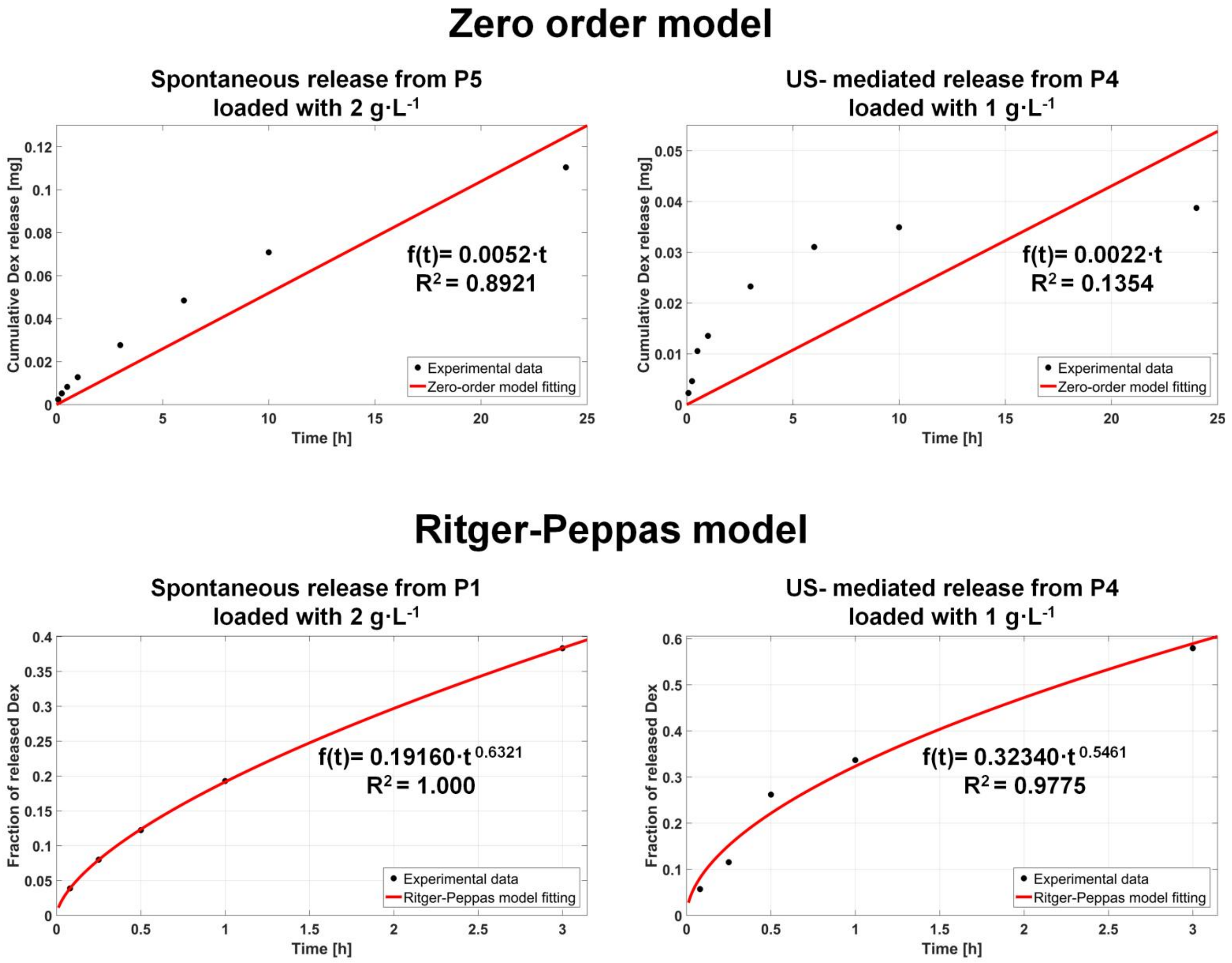

Section S1: Synthesis of monomers

2-n-Propyl-2-oxazoline (1)

2-n-Propyl-2-oxazoline (nPropOx) was prepared according to the procedure for the first time reported by Witte and Seeliger[6] adopted with slight modifications. In a round bottom flask, butyronitrile (55.6 g, 0.805 mol) and zinc acetate (2.68 g, 0.015 mol) were heated to 100 °C. Next, 2-aminoethanol (44.7 g, 0.731 mol) was added dropwise into the reaction mixture. The reaction mixture was stirred at 130 °C for 48 hrs to yield dark brown liquid. The colourless nPropOx was obtained from the reaction mixture by fraction distillation. The third fraction of the product was dried over KOH and CaH_2 and distilled prior to use (25.6 g, yield 31%, bp^{30mBar} =55 °C, lit. bp^{40mBar} = 68 °C [7], purity 96 % (HPLC)).

^{1}H NMR (400 MHz, $CDCl_3$, δ) 0.97 (t, 3H; *CH_3*-CH_2-CH_2), 1.65 (m, 2H; CH_3-*CH_2*-CH_2), 2.25 (m, 2H; CH_3-CH_2-*CH_2*), 3.82 (t, 2H; CH_2-N), 4.21 (t, 2H; CH_2-O).

2-Butyl-2-oxazoline (2)

2-Butyl-2-oxazoline (ButOx) was prepared according to the procedure for the first time reported by Witte and Seeliger[6] adopted with slight modifications. Briefly, in a round bottom flask the valeronitrile (23.85 g, 0.287 mol) and zinc acetate (0.96 g, 0.005 mol) were heated to 130°C. 2-Aminoethanol (16 g, 0.262 mol) was subsequently added dropwise into the reaction mixture. Reaction was stirred under the argon atmosphere at 130°C for 20 hrs followed by addition of dichloromethane (50 ml). After the washing with brine and water, the organic layer was dried over anhydrous sodium sulfate. Sodium sulfate was filtered off and solvent was evaporated. Brownish liquid was dried over KOH for two days and distilled over CaH_2 to yield ButOx as colourless liquid (17.22 g, yield 47%, bp^{14mbar}= 57 °C, lit. $bp^{16\ mbar.}$= 61 °C [8], purity 93 % (HPLC)).

^{1}H NMR (400 MHz, $CDCl_3$, δ) 0.85 (t, 3H, *CH_3*-CH_2-CH_2-CH_2-), 1.3 (m, 2H; CH_3-*CH_2*-CH_2-CH_2-), 1.54 (m, 2H; CH_3-CH_2-*CH_2*-CH_2-), 2.19 (t, 2H; CH_3-CH_2-CH_2-*CH_2*-), 3.74 (t, 2H; CH_2-N), 4.13 (t, 2H; CH_2-O).

2-(3-Butenyl)-2-oxazoline (3)

2-(3-Butenyl)-2-oxazoline (EnOx) was synthesized in two steps according to a procedure described previously for bis(2-oxazolines)[9]. First, 4-pentenoyl chloride (26.85 g, 0.226 mol) dissolved in dichloromethane (300 mL) was added dropwise to aqueous KOH (38.12 g, 0.679

mol) with 2-chloroethylamine hydrochloride (31.52 g, 0.272 mol) cooled in ice bath. After addition of 4-pentenoyl chloride solution, the reaction mixture was stirred overnight at room temperature. Organic layer was separated, washed with brine and water and dried over anhydrous sodium sulfate. Dichloromethane was evaporated and the obtained liquid yellowish *N*-(2-chloroethyl)pent-4-enamide (yield 33.68 g, 92%) was used for second step without purification. The intermediate product was dissolved in 0.1 M methanolic KOH (250 mL) and refluxed for 6 hrs. Product was dissolved in chloroform and residual KCl was filtered off. Chloroform was evaporated and yellowish product was distilled over CaH_2 and stabilized with 2,6-di-*tert*-butyl-4-methoxyphenol to give a EnOx as a colourless liquid (yield 13.64 g, 52 %, bp^{8mbar}= 47 °C, lit. bp^{20mBar}= 67 °C[10], purity 97 % (HPLC)).

^{1}H NMR (400 MHz, $CDCl_3$, δ, ppm) 2.38 (s, 4H, CH_2=CH-CH_2-CH_2-), 3.82 (t, 2H; CH_2-O), 4.22 (t, 2H; CH_2-N), 5.00-5.09 (m, 2H, CH_2=CH-), 5.80-5.89 (m, 1H, CH_2=*CH*-).

References:

(1) Janas, C.; Mostaphaoui, Z.; Schmiederer, L.; Bauer, J.; Wacker, M. G. *Int. J. Pharm.* **2016**, *509* (1-2), 197–207.

(2) Yang, J.; Yan, J.; Zhou, Z.; Amsden, B. G. *Biomacromolecules* **2014**, *15*, 1346–1354.

(3) Wang, Q.; Jiang, J.; Chen, W.; Jiang, H.; Zhang, Z.; Sun, X. *J. Control. Release* **2016**, *230*, 64–72.

(4) Nidhi, K.; Indrajeet, S.; Khushboo, M.; Gauri, K.; Sen, D. J. *Int. J. Drug Dev. Res.* **2011**, *3* (10), 26–33.

(5) He, Z.; Schulz, A.; Wan, X.; Seitz, J.; Bludau, H.; Alakhova, D. Y.; Darr, D. B.; Perou, C. M.; Jordan, R.; Ojima, I.; Kabanov, A. V; Luxenhofer, R. *J. Control. Release* **2015**, *208*, 67–75.

(6) Witte, H.; Seeliger, W. *Justus Liebigs Ann. Chem.* **1974**, *6* (6), 996–1009.

(7) Salzinger, S.; Huber, S.; Jaksch, S.; Busch, P.; Jordan, R.; Papadakis, C. M. *Colloid Polym. Sci.* **2012**, *290*, 385–400.

(8) Seo, Y.; Schulz, A.; Han, Y.; He, Z.; Bludau, H.; Wan, X.; Tong, J.; Bronich, T. K.; Sokolsky, M.; Luxenhofer, R.; Jordan, R.; Kabanov, A. V. *Polym. Adv. Technol.* **2015**, *26*, 837–850.

(9) Néry, L.; Lefebvre, H.; Fradet, A. *Macromol. Chem. Phys.* **2003**, *204*, 1755–1764.

(10) Gress, A.; Vo, A.; Schlaad, H.; June, R. V; Re, V.; Recei, M.; August, V. *Macromol. Rapid Commun.* **2007**, *40*, 7928–7933.

Figure S9. **^{1}H NMR spectrum of 2-n-propyl-2-oxazoline in $CDCl_3$.**

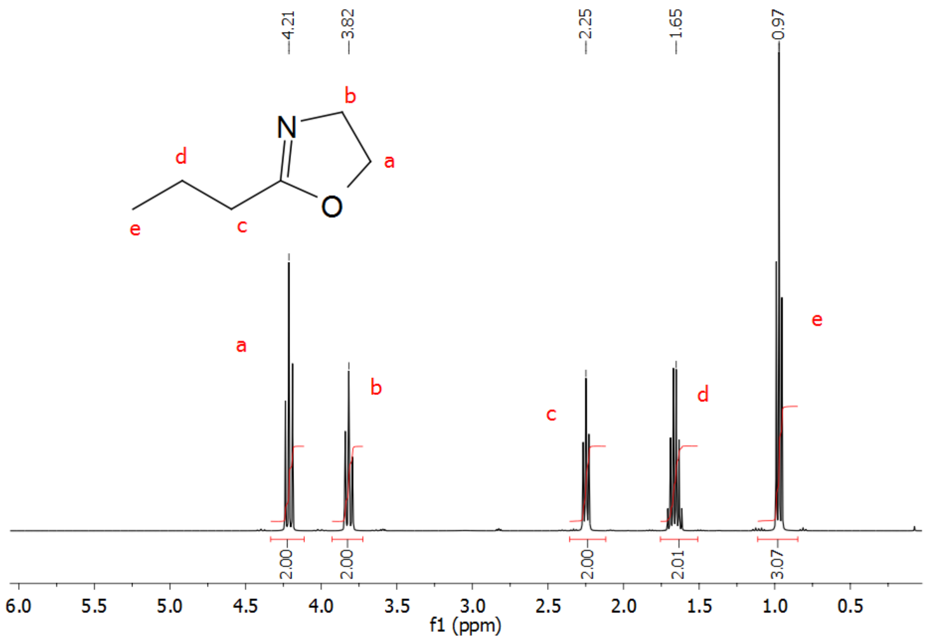

Figure S10. **^{1}H NMR spectrum of 2-butyl-2-oxazoline in $CDCl_3$.**

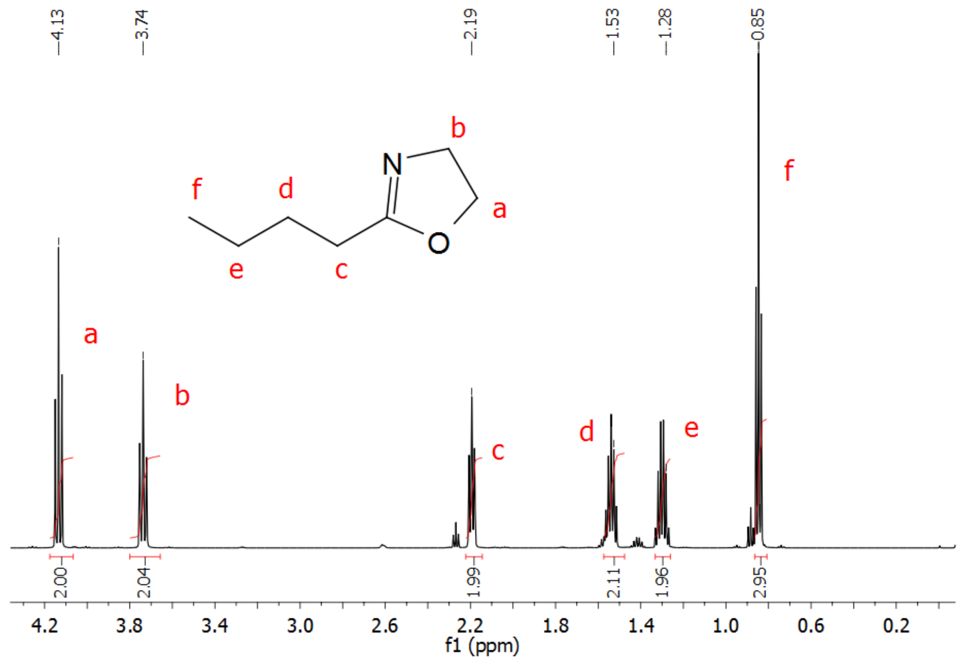

Figure S11. ^{1}H NMR spectrum of 2-2-(3-butenyl)-2-oxazoline in $CDCl_3$.

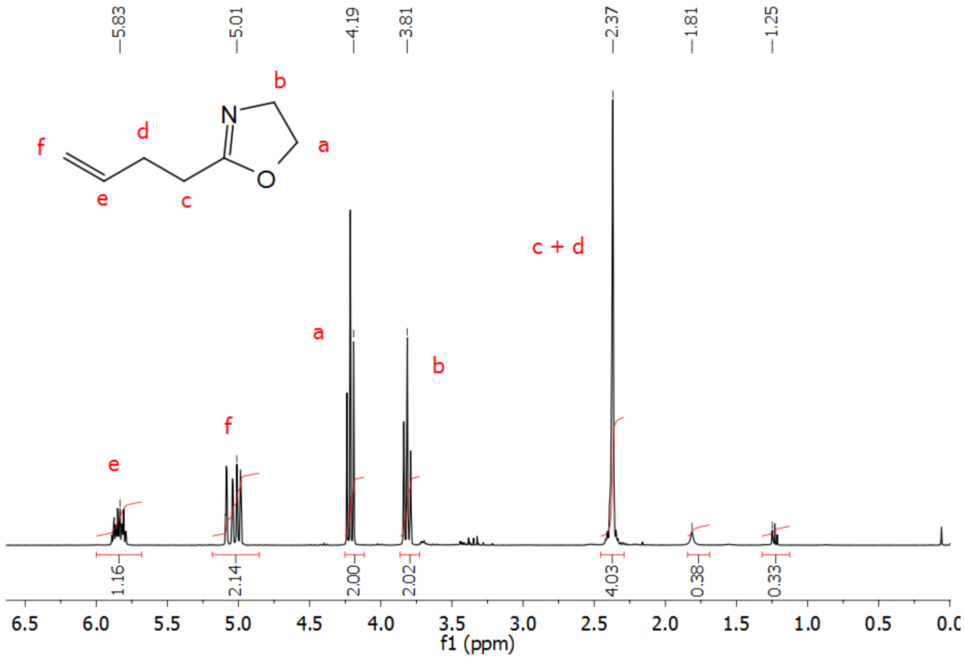

Figure S12. Polymerization reaction scheme of diblock copolymer P1 and P2.

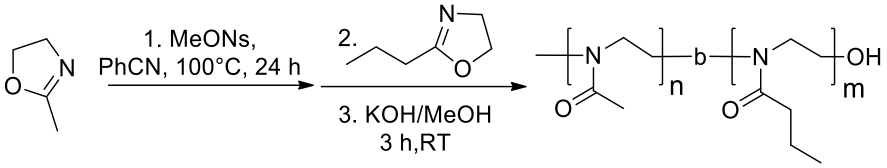

P1 n = 160 , m = 40

P2 n = 40 , m = 60

Figure S13. ^{1}**H NMR spectrum of polymer P1 in CDCl$_3$.**

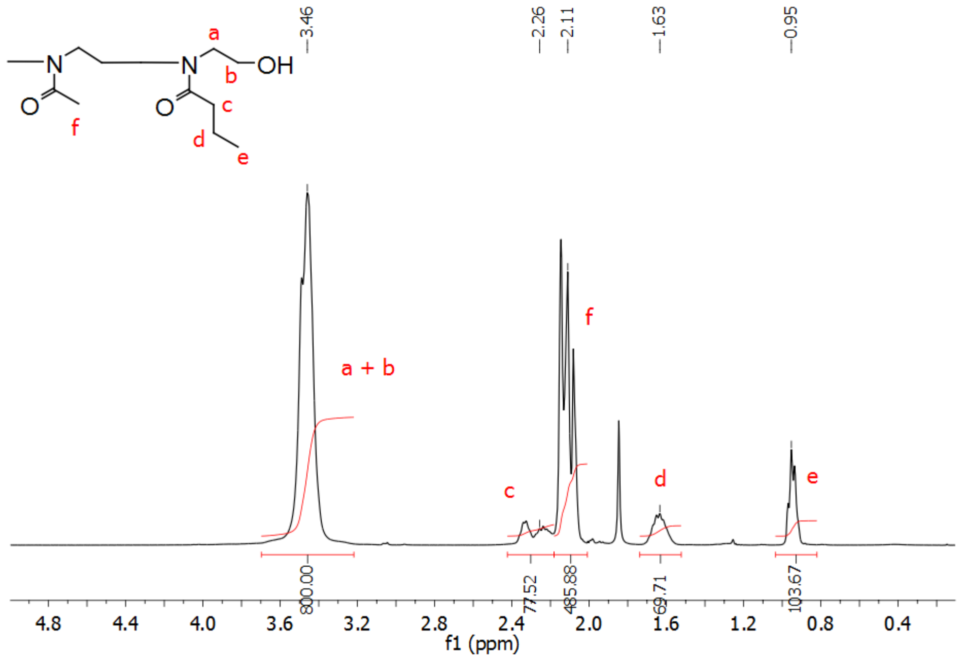

Figure S14. ^{1}**H NMR spectrum of polymer P2 in CDCl$_3$.**

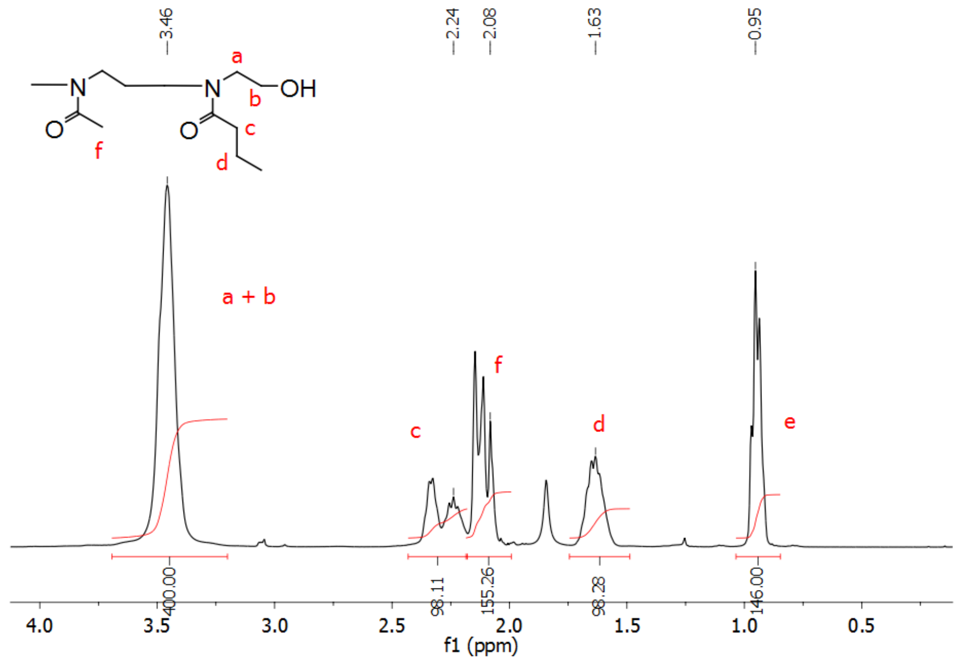

Figure S15. Polymerization reaction scheme of diblock copolymer P3 and P4

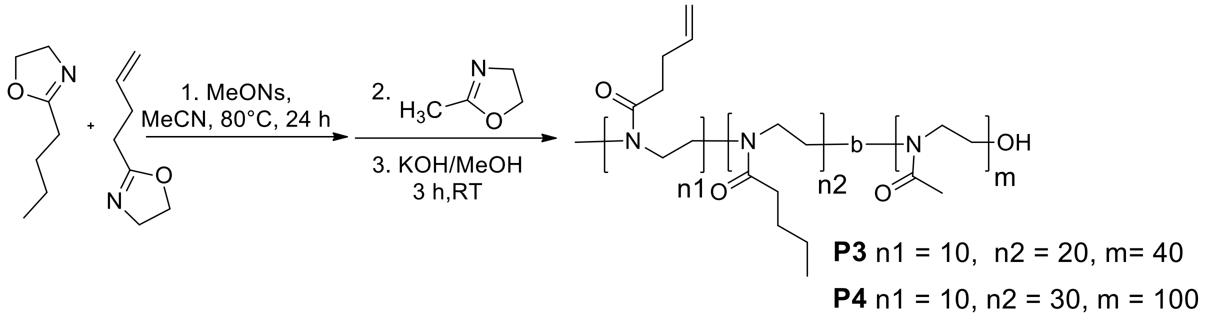

Figure S16. ^{1}H NMR spectrum of polymer P3 in CDCl3.

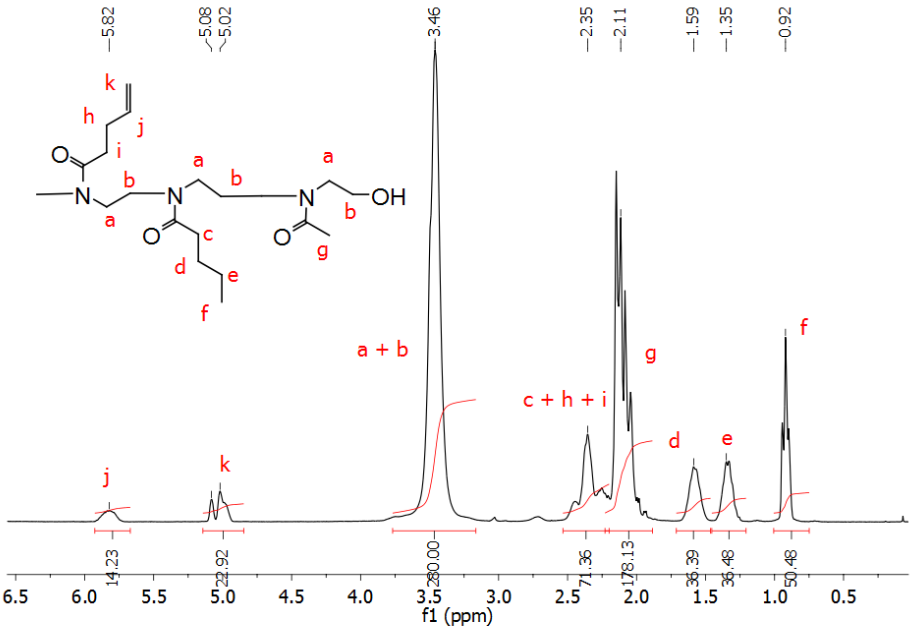

Figure S17. ^{1}H NMR spectrum of polymer P4 in $CDCl_3$.

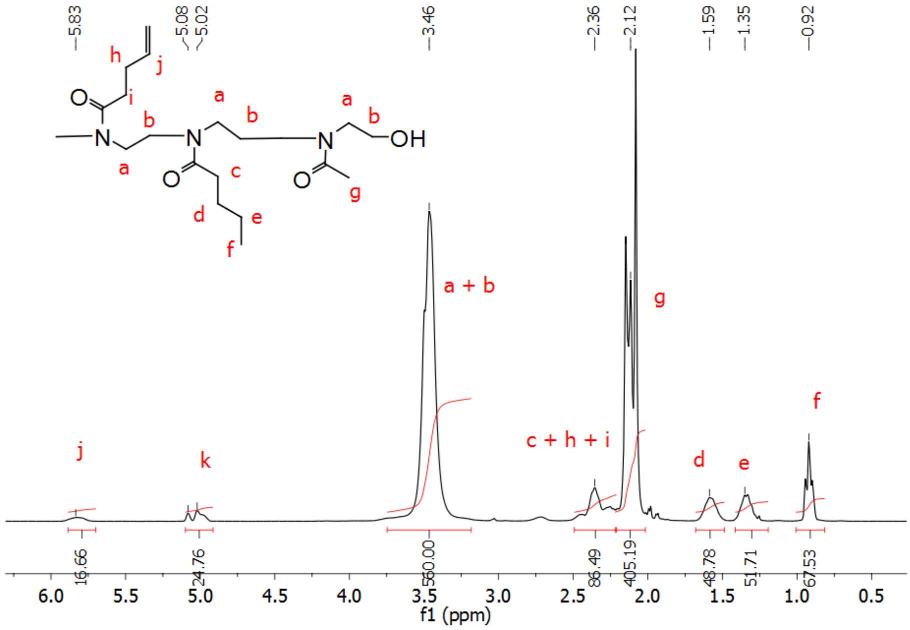

Figure S18. Polymerization reaction scheme of triblock copolymer P5.

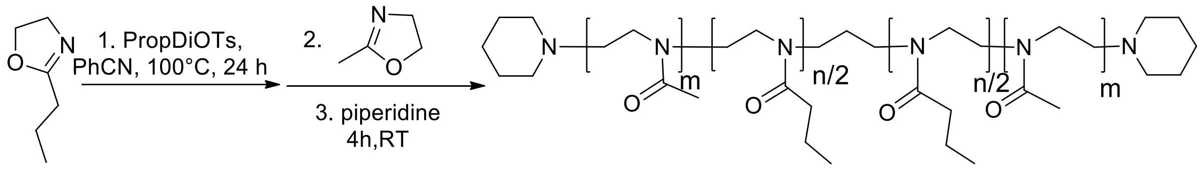

P5 n = 145, m = 25

Figure S19. ^{1}H NMR spectrum of polymer P5 in deuterium oxide.

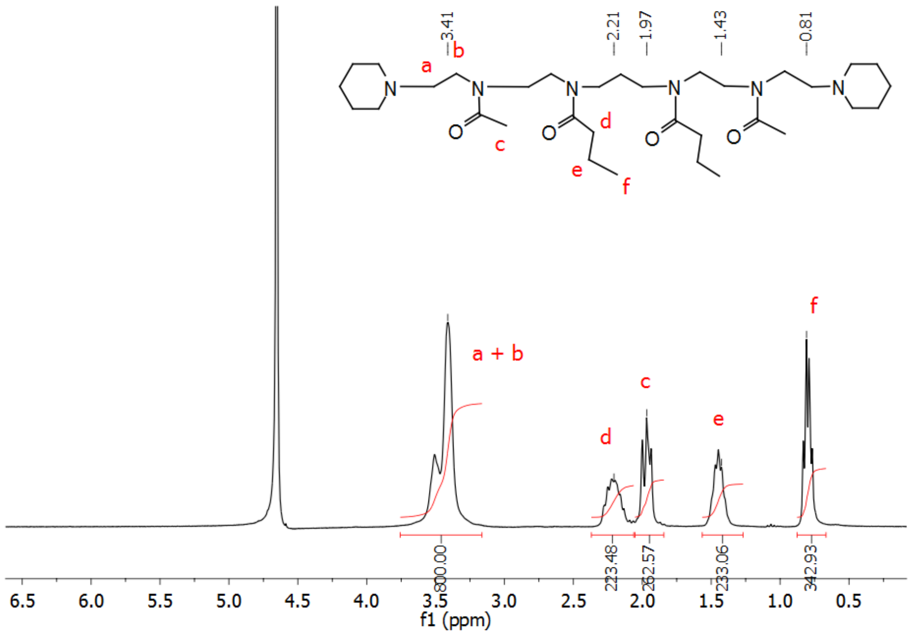

Figure S20. SEC elugrams of the prepared copolymers.

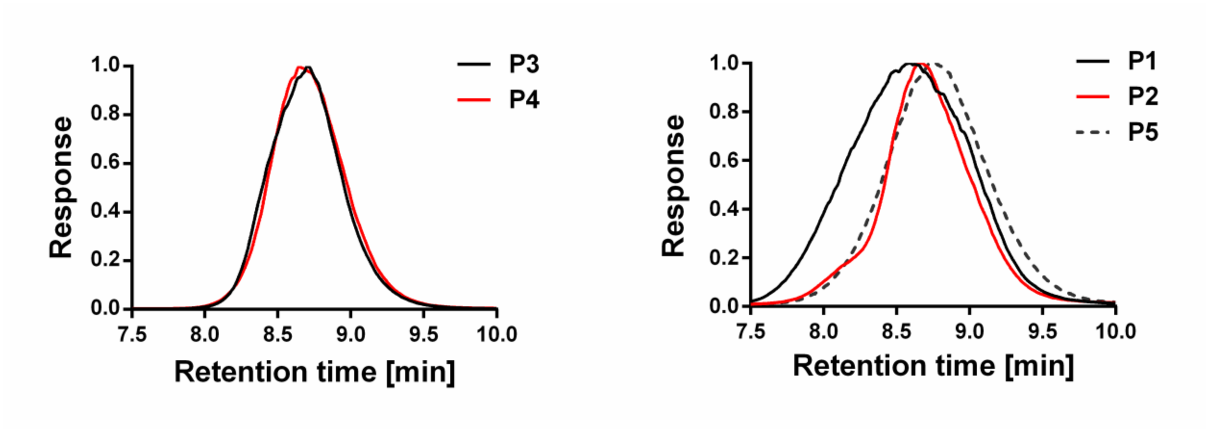

Supplement: Supplementary file 1 — Supplementary Information [file 41598_2018_28140_MOESM1_ESM.pdf]
